# Low Shear Stress Promotes Atherosclerosis by Mediating Pathological Accumulation of Endothelial Lipid Droplets via the KLF4/TFEB/ATP1A1 Axis

**DOI:** 10.3390/jcdd13050213

**Published:** 2026-05-15

**Authors:** Yi Shi, Ya-Nan Tan, Li-Da Wu, Li-Guo Wang, Yue Gu, Wen-Ying Zhou, Meng-Qian Shao, Jun-Xia Zhang

**Affiliations:** 1 Department of Cardiology, Nanjing First Hospital, Nanjing Medical University, Nanjing 210006, China; shiyi@stu.njmu.edu.cn (Y.S.); nan_0723@stu.njmu.edu.cn (Y.-N.T.); lidawu@stu.njmu.edu.cn (L.-D.W.); guyue1004@njmu.edu.cn (Y.G.); mkwenying929@163.com (W.-Y.Z.); shaomengqian@163.com (M.-Q.S.); 2 Department of Cardiology, Sir Run Run Shaw Hospital, Zhejiang University School of Medicine, Hangzhou 310016, China; 12418523@zju.edu.cn

**Keywords:** low shear stress, lipid droplets, atherosclerosis, lipophagy, KLF4

## Abstract

Background: Atherosclerosis preferentially develops at arterial regions exposed to low shear stress (LSS), highlighting the critical role of local hemodynamic forces in disease initiation and progression. Emerging evidence indicates that endothelial lipid metabolism is a key determinant of vascular homeostasis; however, whether LSS directly regulates endothelial lipid droplets’ (LDs) dynamics remains unclear. In particular, the mechano-transduction pathways linking shear stress to lysosome-mediated lipid processing within the endothelium have yet to be defined. Methods: Complementary in vitro flow systems and in vivo atheroprone models were employed to examine the effects of LSS on endothelial lipid metabolism. Endothelial LDs accumulation, lysosome-dependent lipophagy, and atherosclerotic lesion development were systematically assessed under LSS conditions. Mechanistically, molecular profiling and rapamycin-mediated functional rescue were conducted to delineate the role of the KLF4/TFEB/ATP1A1 signaling axis in LSS-induced impairment of lysosome-dependent lipophagy. Results: We found that LSS induced pathological accumulation of LDs in vascular endothelial cells, accompanied by a marked suppression of lysosome-dependent lipophagy. Elucidation of the mechanism showed that LSS downregulated the shear-responsive transcription factor KLF4, resulting in aberrant phosphorylation of transcription factor EB (TFEB) and impaired TFEB nuclear translocation. Consequently, the TFEB transcriptional program governing lysosomal function was disrupted, including reduced expression of the TFEB target ATP1A1, leading to defective lysosomal acidification and blockade of lipid autophagic flux. Restoration of the KLF4/TFEB/ATP1A1 axis reactivated lipophagy, alleviated endothelial lipid burden, and significantly attenuated atherosclerotic lesion development. Conclusions: Our findings demonstrate that disruption of the KLF4/TFEB/ATP1A1 signaling pathway mediates LSS-induced impairment of endothelial lipophagy, thereby driving pathological LDs accumulation. This highlights the potential of restoring this axis as a therapeutic strategy to attenuate atherosclerotic progression.

## 1. Introduction

Atherosclerosis (AS) is a spatially heterogeneous disease that preferentially develops at specific regions of the arterial tree such as branch points and curvatures where laminar blood flow is disturbed [1]. Extensive experimental and clinical evidence has established that local hemodynamic forces play a critical role in shaping endothelial phenotypes and determining vascular susceptibility to disease [2]. In particular, low shear stress (LSS), occurring at branch points and regions of disturbed flow, is strongly associated with the development of atheroprone vascular regions. Conversely, physiological shear stress (PSS) helps maintain endothelial homeostasis and exerts atheroprotective effects [3]. As the luminal surface of blood vessels, endothelial cells (ECs) function as the primary sensors of mechanical forces derived from blood flow. These cells convert fluid shear stress into intracellular biochemical signals through endothelial mechano-transduction, thereby regulating endothelial structure and function. This process suggests that LSS may serve as a primary mechano-metabolic regulator, fundamentally influencing the initiation and progression of atherosclerotic lesions by predisposing the endothelium to chronic metabolic dysregulation.

Beyond their fundamental roles as physical barriers and structural scaffolds, ECs are increasingly recognized as metabolically active gatekeepers of vascular homeostasis [4]. Emerging evidence underscores that shear stress (SS) serves as a primary determinant in orchestrating endothelial metabolic reprogramming, encompassing glycolysis, mitochondrial bioenergetics, and redox proteostasis, which collectively sustain endothelial integrity under diverse hemodynamic regimes [5]. Specifically, it remains to be established how hemodynamic regimes dictate the spatiotemporal dynamics of endothelial LDs beyond the influence of systemic hyperlipidemia, as well as the mechanisms by which such localized metabolic reprogramming drives atherosclerotic progression.

The maintenance of LDs homeostasis relies largely on lysosome-dependent autophagy, a process termed lipophagy, which facilitates the mobilization and degradation of intracellular lipids [6]. Accumulating evidence indicates that impairment of lysosomal function and autophagic flux contributes to lipid accumulation [7,8]. Despite these advances, the potential for hemodynamic forces to govern endothelial lipophagy by modulating lysosomal acidification remains a critical missing link in vascular mechanobiology. Accordingly, the precise organelle-level mechanisms underlying this regulatory process have yet to be fully delineated.

Krüppel-like factor 4 (KLF4) is a SS-responsive transcription factor essential for maintaining endothelial homeostasis and vascular integrity [9]. Evidence indicates that KLF4 is preferentially induced by PSS and exerts anti-inflammatory and anti-atherogenic effects by orchestrating endothelial gene programs involved in inflammation, redox balance, and metabolism, while endothelial KLF4 deficiency exacerbates vascular inflammation and accelerates atherosclerotic lesion progression [10]. Given its broad effects on endothelial function and metabolism, KLF4 may influence cellular responses to SS and the metabolic regulation through indirect modulation of intracellular signaling pathways [11]. Transcription factor EB (TFEB) is a pivotal regulator of lysosomal biogenesis and autophagy through phosphorylation-mediated regulation of its subcellular localization [12]. However, the direct molecular coupling between KLF4 and TFEB remains poorly defined; specifically, it remains unclear whether KLF4 acts as a hemodynamically responsive ‘regulatory switch’ that modulates the phosphorylation status of TFEB, thereby regulating its nuclear translocation and functional activation, so as to participate in the regulation of endothelial lipid metabolism under LSS conditions.

In this study, we demonstrate that LSS triggers the pathological accumulation of LDs within the vascular endothelium by impairing lysosome-dependent lipophagy. We further identify the KLF4/TFEB/ATP1A1 signaling axis as a critical mechano-metabolic bridge linking biomechanical cues to endothelial lipid metabolism. Furthermore, pharmacological intervention with RAPA, or genetic restoration of the KLF4/TFEB axis, effectively bypasses LSS-induced signaling blockade, thereby restoring lysosomal function and re-establishing endothelial metabolic fitness. Collectively, these findings uncover a previously unrecognized mechanism by which RAPA acts as a site-specific metabolic stabilizer, and establish the KLF4/TFEB/ATP1A1 axis as a key nexus between biomechanical signaling and endothelial lipid homeostasis, highlighting a promising therapeutic strategy for alleviating AS by targeting biomechanically driven lipid metabolic dysfunction.

## 2. Materials and Methods

### 2.1. Animal Models

The male Apolipoprotein E knockout (ApoE^−/−^) mice and C57B6/J mice, at 8 weeks of age, were obtained from the Nanjing Medical University Animal Laboratory. They were raised in a temperature of 20–24 °C under a 12 h light–dark cycle, provided with unlimited food and water throughout the experiment. The study protocol was approved by the Animal Care Committees of the Laboratory Animal Centre and Nanjing First Hospital. Animals were randomly assigned to experimental groups. A simple randomization procedure was performed using a random number generator (Excel 2019, Microsoft, Redmond, DC, USA) to allocate each mouse to either the Sham group, Model group, or Treatment group. First, the design of AS included two groups: one group of ApoE^−/−^ Chow mice fed a standard chow diet (open standard diet with 15 Kcal% fat, Research Diets, D11112201, New Brunswick, NJ, USA) and ApoE^−/−^ HFD group fed a HFD (40% kcal from fat plus 1.25% cholesterol, Research Diets, D12108C) for 12 weeks. Partial ligation was achieved by permanently tying off three of the four major distal branches of the LCA with 6-0 silk sutures: the left external carotid artery (ECA), the internal carotid artery (ICA), and the occipital artery (OA). The superior thyroid artery (STA) was left intact, while the right common carotid artery (RCA) was left untouched to serve as the internal control [13]. In addition, we administered Rapamycin (RAPA) (HY-10219, MedChemExpress, Monmouth Junction, NJ, USA) through intraperitoneal injection in ApoE^−/−^ HFD mice at a dosage of 5 mg/(kg·d) for 4 weeks, with the ApoE^−/−^ chow group receiving an equal volume of phosphate-buffered saline (PBS) (G4202, Servicebio, Wuhan, China) solution. For subsequent analyses, the left common carotid artery (LCA) was dissected and harvested from the region spanning between the aortic arch and the ligation sites (distal to the carotid bifurcation). The right common carotid artery (RCA), serving as the control, was collected from the segment between the right subclavian artery and the carotid bifurcation. Inclusion and exclusion criteria were established a priori. Mice were included in the study only if they successfully underwent the surgical procedure and survived until the experimental endpoint. Animals were excluded from the analysis if they died during or immediately after surgery/injection. All procedures were approved by the Institutional Animal Care and Use Committee (IACUC-2007012) of Nanjing Medical University and were in accordance with the Guide for the Care and Use of Laboratory Animals (National Academies Press, 2011) [14].

### 2.2. Bioinformatics Analysis

To identify key regulatory genes mediating endothelial lipid metabolism under pathological shear stress, we performed an integrated bioinformatics analysis using three independent datasets from the Gene Expression Omnibus (GEO) database, encompassing both in vitro and in vivo models. Specifically, we utilized: (1) GSE45225 [14], involving HUVECs exposed to low shear stress (LSS, 1 dyne/cm^2^) compared to physiological shear stress (PSS, 10 dyne/cm^2^) in a laminar flow bioreactor; (2) GSE103672 [15], a longitudinal transcriptomic profiling of HUVECs subjected to oscillatory shear stress (OS, 0.5 ± 5 dyn/cm^2^) versus pulsatile shear stress (PS, 12 ± 5 dyn/cm^2^); and (3) GSE197366 [16], representing endothelial-enriched RNAs from the carotid arteries of young (8-week-old) male C57BL/6 mice subjected to partial carotid ligation (PCL)-induced disturbed flow (DF). To maintain biological consistency across species, mouse gene symbols from GSE197366 were converted to their human orthologs via humanization transformation. For each dataset, differentially expressed genes (DEGs) were identified using the criteria of |log_2_ Fold Change| > 1 and *p* < 0.05, with distributions visualized via volcano plots. To focus on lipid-related mechanisms, a gene set associated with “lipid droplets” (LDs) was independently retrieved from the GeneCards database (https://www.genecards.org). Finally, the three DEG sets and the GeneCards LD-related gene set were intersected using Venn diagram analysis. This integrated screening strategy identified KLF4 as a central LD-regulatory gene responsive to multiple patterns of pathological flow, which was selected for subsequent experimental validation.

### 2.3. Reagents

The primary antibodies employed in this study including anti-P62 (A19700, ABclonal Technology, Wuhan, China), anti-LC3B (A19665, ABclonal Technology), anti-GAPDH (60004-1-Ig, Proteintech, Tokyo, Japan), anti-VWF (GB11020, Servicebio), anti-LAMP1 (sc-20011, Santa Cruz Biotechnology, Dallas, TX, USA), anti-TFEB (83010, Cell Signaling Technology, Danvers, MA, USA), anti-Phospho-TFEB (37681, Cell Signaling Technology), anti-ATP1A1 (14418-1-AP, Proteintech) and anti-LC3B (sc-271625, Santa Cruz Biotechnology). Western blot and fluorescent secondary antibodies were all purchased from Servicebio including HRP-labeled goat anti-rabbit IgG (GB23303), HRP-labeled goat anti-mouse IgG (GB23301), Alexa Fluor 488-labeled goat anti-rabbit IgG (GB25303), Alexa Fluor 488-labeled goat anti-mouse IgG (GB25301), Cy3-labeled goat anti-mouse IgG (GB21301) and Cy3-labeled goat anti-rabbit IgG (GB21303). Rapamycin (HY-10219) was purchased from MedChemExpress. Enhanced chemiluminescence (ECL) reagent was obtained from Trans Gen Biotech (Beijing, China, DW101-02). DAPI (36308ES20, YEASEN, Shanghai, China) was also used. All the other chemicals involved were of experimental level and purchased from standard commercial suppliers.

### 2.4. Tissue Sampling and Histological Staining

Mice were euthanized under deep anesthesia achieved through an intraperitoneal injection of ketamine or xylazine, followed by intracardiac perfusion with phosphate-buffered saline (PBS). The aorta and regions of the aortic valve were collected and fixed overnight in 4% paraformaldehyde. The tissues were then processed for either paraffin embedding or frozen sectioning (5 μm thick). The paraffin sections were used for Masson, H&E, and Movat staining and the frozen sections were used for Oil Red staining. After staining, the samples were mounted and observed under a light microscope (U-TR30-2, OLYMPUS, Tokyo, Japan) for imaging and documentation.

### 2.5. Cell Culture

Human umbilical vein endothelial cells (HUVECs) were sourced from the Cell Bank of the Chinese Academy of Sciences in Shanghai, China. The cells were maintained in ECM medium (ScienCell, Carlsbad, CA, USA, Cat No.1001) enriched with 10% matched fetal bovine serum (FBS), 1% endothelial cell growth supplements (ECGs), and 1% P/S solution, under standard culture conditions of 37 °C with 5% CO_2_. In the cell model, RAPA was used at a concentration of 100 nmol/L for 6 h before dealing with shear stress, while the control group received an equal volume of PBS (G4202, Servicebio).

### 2.6. Low Shear Stress Simulation Device

HUVECs were seeded onto fibronectin-coated slides (μ-Slide I0.8 Luer, ibidi) and cultured for 24 h. They were then exposed to LSS (2 dynes/cm^2^) or physiological shear stress (PSS) (15 dynes/cm^2^) for 12 h in a parallel plate flow chamber system (ibidi Pump System,10902-S; ibidi GmbH, Gräfelfing, Germany).

### 2.7. Immunofluorescence Staining

HUVECs, tissue sections, or vascular samples were washed three times with PBS (G4202, Servicebio), fixed with 4% paraformaldehyde (G1101, Servicebio) for 10 min, and then permeabilized with 0.1% Triton X-100 (GC204003, Servicebio) for 30 min at around 37 °C. The samples were subsequently blocked with 5% BSA (A1933, Sigma-Aldrich, St. Louis, MO, USA). They were then incubated overnight at 4 °C with primary antibodies, including anti-VWF (1:200), anti-LC3B (1:100), anti-P62 (1:100), and anti-LAMP1 (1:100). The samples were also incubated with LDs dye (1:10,000, C2053S, Beyotime, Shanghai, China) and LysoSensor Blue DND-167 (1:10,000, L7533, Thermo Fisher, Waltham, MA, USA) at room temperature for 1 h. Afterward, the corresponding secondary fluorescent antibodies were applied at room temperature in the dark for 1 h. DAPI (36308ES20, YEASEN) was used for nuclear locating. Images were acquired using confocal microscopy (Zeiss LSM 880, Oberkochen, Germany).

### 2.8. Immunoblotting Analysis

By employing an in situ intimal flushing technique, total proteins were selectively extracted from the vascular endothelium using ice-cold RIPA buffer (G2002, Servicebio) supplemented with protease and phosphatase inhibitors (G2006 and G2007, Servicebio). This approach allowed for the rapid and precise harvest of endothelial components with minimal contamination from the underlying vascular layers. The protein samples were separated by sodium dodecyl sulfate–polyacrylamide gel electrophoresis (SDS-PAGE, Yeasen Biotech, Beijing, China) and transferred to a polyvinylidene fluoride (PVDF) membrane (ISEQ00005, Millipore, Burlington, MA, USA). The primary and secondary antibodies used were anti-p62 (1:1000), anti-LC3B (1:1000), anti-GAPDH (1:10,000), anti-TFEB (1:1000), anti-phospho-TFEB (1:1000), anti-ATP1A1 (1:1000), and anti-LAMP1 (1:500). Protein signals were detected using ECL chemiluminescence reagent and HRP-conjugated anti-rabbit or anti-mouse IgG antibodies (1:10,000). The protein molecular weight marker (MP-102-AA) was obtained from Vazyme (Nanjing, China).

### 2.9. ELISA

First, mouse blood samples were collected and kept at room temperature for 20 min to allow clot formation. Next, the samples were placed in a pre-chilled centrifuge and centrifuged at 5000 rpm for 10 min at 4 °C to separate the serum. Then, the serum supernatant was carefully transferred to a new centrifuge tube using a pipette. Finally, total cholesterol (TC) and triglyceride (TG) levels were measured in triplicate using an ELISA kit (ABclonal Technology) according to the manufacturer’s instructions.

### 2.10. RT-qPCR

Endothelial total RNA was isolated via in situ intimal flushing as previously described [17]. Following heparinized saline perfusion, the carotid lumen was flushed with QIAzol lysis buffer (from the miRNeasy Mini Kit, Qiagen, Hilden, Germany) for approximately 1 s to selectively lyse the endothelium while preserving the media and adventitia, followed by RNA purification using the same kit optimized for micro-scale recovery. Sample purity and concentration were determined via NanoDrop spectrophotometer (Thermo Scientific, Wilmington, DE, USA), with endothelial enrichment confirmed by high CD31 and negligible α-SMA expression through RT-qPCR. A total of 500–1000 ng RNA was used for reverse transcription using HiScript III RT SuperMix for qPCR (+gDNA wiper) (R423, Vazyme). The qPCR reactions were performed in duplicate with ChamQ Universal SYBR qPCR Master Mix (Q711, Vazyme) on a QuantStudio 3 Real-Time PCR System (Applied Biosystems, Foster City, CA, USA) according to the manufacturer’s protocol. After normalization to GAPDH, mRNA levels were determined via the 2^−ΔΔCt^ method. The specific primer sequences used for real-time RT-PCR analysis are listed in Supplementary Table S1. All primer sequences were designed using the NCBI website (https://www.ncbi.nlm.nih.gov/) and have been validated for specificity via the Primer-Blast tool.

### 2.11. Statistical Analysis

This study used GraphPad Prism 8.0 software (GraphPad Software, San Diego, CA, USA) for statistical analysis of data. First, data normality was tested. Quantitative data with a normal distribution were expressed as mean ± standard error of the mean (SEM). For data without a normal distribution, results were expressed as median (interquartile range) and were log-transformed before further analysis. A one-sample t-test was used to determine whether the mean of a sample was significantly different from a known population mean (such as 0), and an unpaired two-tailed Student’s t test was used for comparisons between two independent groups. For multiple-group comparisons, one-way analysis of variance (ANOVA) followed by Bonferroni’s post hoc test was applied for single-factor designs, whereas two-way ANOVA followed by Tukey’s multiple comparison test was used for two-factor designs. All tests were two-tailed, and a *p* < 0.05 was considered statistically significant.

## 3. Results

### 3.1. Low Shear Stress Promotes Endothelial Lipid Droplet Accumulation and Metabolic Dysfunction In Vivo and In Vitro

**Figure 1:**
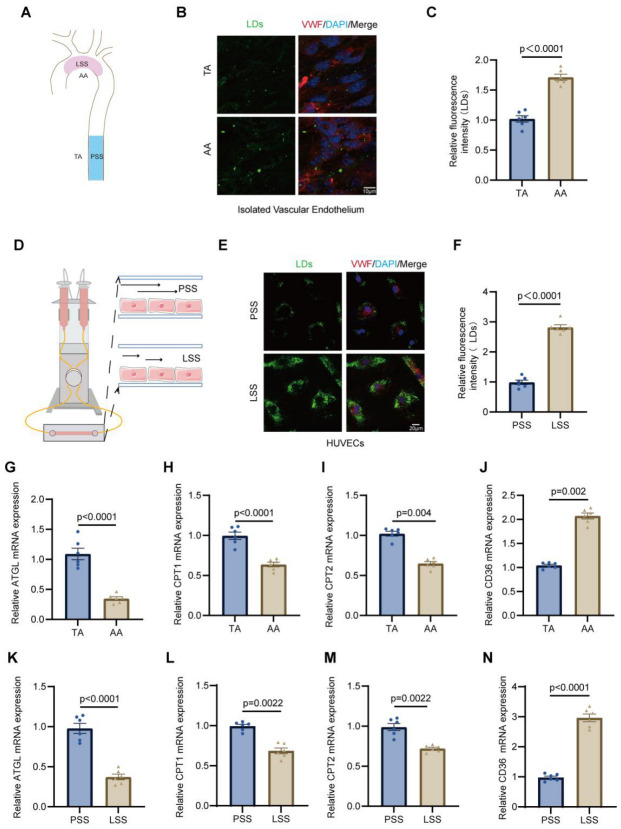
Low shear stress induces lipid droplet accumulation and disrupts lipid metabolic homeostasis in endothelial cells. (**A**), Schematic illustration of blood flow patterns at the inner curvature of the aortic arch (AA) and thoracic aorta (TA). (**B**), Representative fluorescence images of lipid droplets (LDs) in endothelial cells from the AA and TA regions isolated from ApoE^−^/^−^ mice fed with high fat diet (HFD). (**C**), Quantification of relative LD fluorescence intensity in endothelial cells from the AA and TA regions of ApoE^−^/^−^ HFD mice. (**D**), Schematic diagram of the in vitro flow system used to expose human umbilical vein endothelial cells (HUVECs) to low shear stress (LSS) or physiological shear stress (PSS). (**E**), Representative immunofluorescence images of lipid droplets (LDs, green), the endothelial marker von Willebrand factor (VWF, red), and nuclei (DAPI, blue) in HUVECs subjected to LSS or PSS. (**F**), Quantification of LDs fluorescence intensity in HUVECs exposed to LSS or PSS. (**G**–**J**), RT-qPCR analysis of mRNA expression of ATGL, CPT1, CPT2, and CD36 in endothelial cells isolated from the TA and AA regions of HFD ApoE^−^/^−^ mice. (**K**–**N**), RT-qPCR analysis of mRNA expression of ATGL, CPT1, CPT2, and CD36 in HUVECs exposed to PSS or LSS. AA, the inner curvature of the aortic arch; TA, thoracic aorta; LD, lipid droplet; HUVEC, human umbilical vein endothelial cell; LSS, low shear stress; PSS, physiological shear stress; ATGL, adipose triglyceride lipase; CPT1/CPT2, carnitine palmitoyltransferase 1 and 2; CD36, cluster of differentiation 36. All data are presented as mean ± SEM. Statistical analyses were performed using unpaired two-tailed Student’s *t* test, as appropriate. *n* = 6 mice per group.

To investigate the association between LSS and endothelial lipid metabolism during AS, we first evaluated atherosclerotic lesion formation in ApoE^−/−^ mice fed a high-fat diet (HFD) (Figure S1). Oil Red O staining revealed a higher plaque area fraction in the inner curvature of the aortic arch (AA) in HFD-fed ApoE^−/−^ mice, a region prone to LSS. In contrast, the thoracic aorta (TA), a region exposed to PSS, showed a lower plaque area fraction (Figure 1A and Figure S1A,B). Fluorescence staining demonstrated pronounced LD accumulation in endothelial cells within AA area compared with the TA in HFD-fed ApoE^−/−^ mice, as well as in HUVECs exposed to LSS in vitro (Figure 1B–F). To further assess lipid metabolic alterations, RT-qPCR analysis was performed. The expression of the lipolytic enzyme adipose triglyceride lipase (ATGL), and the mitochondrial fatty acid transport carnitine palmitoyltransferase 1 and 2 (CPT1 and CPT2) was markedly reduced in the AA compared with the TA (Figure 1G–I). Conversely, the fatty acid uptake protein CD36 (cluster of differentiation 36) was upregulated in the AA (Figure 1J). Consistent expression changes were also detected in HUVECs subjected to LSS compared with PSS (Figure 1K–N). Collectively, these findings indicate that LSS is associated with enhanced endothelial lipid droplet accumulation accompanied by dysregulated lipid metabolic homeostasis, both in vivo and in vitro.

### 3.2. Reduced Lysosomal Lipophagy Drives Lipid Droplet Accumulation Under Low Shear Stress

**Figure 2:**
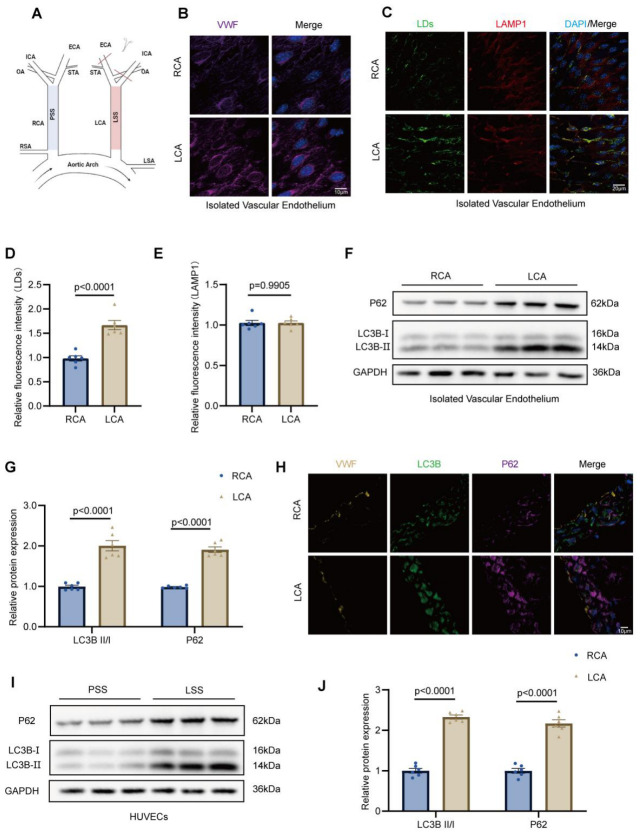
Reduced lysosomal lipophagy drives lipid droplet accumulation under low shear stress. (**A**), Schematic illustration of the partial ligation of the left carotid artery (LCA), with the contralateral right carotid artery (RCA) as control. (**B**), Representative enface immunofluorescence images of endothelial cells stained for von Willebrand factor (VWF, purple) and nuclei (DAPI, blue) in RCA and LCA. (**C**), Representative enface images of lipid droplets (LDs, green), nuclei (DAPI, blue) and LAMP1 (red) in RCA and LCA. (**D**,**E**), Quantification of LDs or LAMP1 fluorescence intensity in RCA and LCA. (**F**), Western blot analysis of p62 and LC3B-II/I levels in proteins isolated from RCA and LCA. (**G**), Quantification of p62 and LC3B-II/I levels from Western blot. (**H**), Representative immunofluorescence images of frozen carotid sections stained for VWF (brown), LC3B (green), and p62 (purple) in RCA and LCA. (**I**), Western blot analysis of p62 and LC3B-II/I levels in HUVECs exposed to low shear stress (LSS) or physiological shear stress (PSS). (**J**), Quantification of Western blot results from HUVECs. LCA, left carotid artery; RCA, right carotid artery; ECA, external carotid artery; ICA, internal carotid artery; STA, superior thyroid artery; OA, occipital artery; RSA, Right Subclavian Artery; LSA, Left Subclavian Artery; LD, lipid droplet; VWF, von Willebrand factor; LAMP1, lysosomal-associated membrane protein 1; P62, sequestosome 1; LC3B, microtubule-associated protein 1 light chain 3 beta; HUVEC, human umbilical vein endothelial cell; LSS, low shear stress; PSS, physiological shear stress. All data are presented as mean ± SEM. Statistical analyses were performed using unpaired two-tailed Student’s *t* test or two-way ANOVA followed by Tukey’s multiple comparison test, as appropriate. *n* = 6 for both cell and animal experiments.

To elucidate the mechanisms by which LSS promotes endothelial LDs accumulation, we established a partial ligation model of the left carotid artery (LCA), with the contralateral right carotid artery (RCA) serving as an internal control (Figure 2A). Enface staining of VWF revealed endothelial architecture in the LCA and RCA (Figure 2B). Then, enface staining of LDs and LAMP1 demonstrated increased LDs deposition in the LCA, whereas LAMP1 expression remained comparable between LCA and RCA (Figure 2C–E). Western blot analysis of isolated vessel proteins confirmed elevated levels of autophagy markers P62 and LC3B-II/I in the LCA compared with the RCA (Figure 2F,G). Analogously, immunofluorescence staining of frozen carotid sections corroborated these findings, showing enhanced p62 and LC3B signals specifically in LCA endothelial cells (Figure 2H). Consistent with the in vivo findings, HUVECs subjected to LSS exhibited increased P62 levels and an elevated LC3B-II/I ratio relative to PSS controls, indicative of impaired autophagic flux under LSS treatment (Figure 2I,J). To further elucidate lysosomal function under conditions of impaired lipophagy, we examined lysosomal markers in both LCA and RCA endothelium, as well as in HUVECs subjected to LSS or PSS. Western blot and enface immunofluorescence analyses demonstrated that LAMP1 expression remained comparable between LCA and RCA, whereas Lyso Tracker fluorescence was markedly diminished in the LCA, indicative of impaired lysosomal acidification (Figure S2A–E). Similarly, in HUVECs, LSS did not alter LAMP1 levels but caused a pronounced reduction in Lyso Tracker intensity relative to PSS (Figure S2F–I). Together, these observations indicate that LSS selectively compromises lysosomal acidification without affecting lysosomal content, thereby suppressing lipophagy and contributing to pathological LD accumulation in endothelial cells.

### 3.3. KLF4 Is Identified as a Central Molecule in Sensing Mechanical Force to Induce Lipid Metabolism Disorders

**Figure 3:**
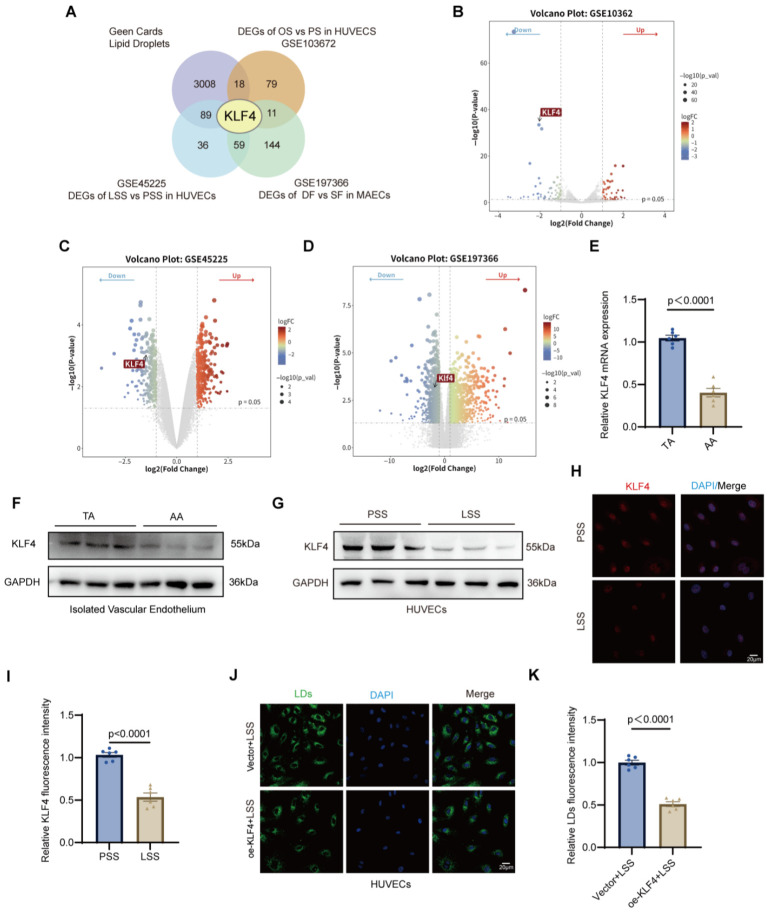
KLF4 as a key mechanosensitive regulator of lipid metabolism under low shear stress. (**A**), Venn diagram illustrating the intersection of differentially expressed genes (DEGs) from three transcriptomic datasets (GSE45225, GSE103672, and GSE197366) and a lipid droplet-related gene set retrieved from GeneCards. (**B**–**D**), Volcano plots showing differentially expressed genes and KLF4 in the GSE45225, GSE103672 and GSE197366. Significantly regulated genes are defined by |log2FC| > 1 and *p* < 0.05. The vertical and horizontal dotted lines indicate the thresholds for significantly regulated genes (|log_2FC| > 1 and *p* < 0.05).The KLF4, is specifically indicated in each dataset. (**E**), RT-qPCR analysis of KLF4 mRNA expression in the aortic arch (AA) and thoracic aorta (TA) of HFD-fed ApoE^−/−^ mice. (**F**), Western blot analysis of KLF4 protein expression in AA and TA regions of HFD-fed ApoE^−/−^ mice. (**G**), Western blot analysis of KLF4 protein expression in HUVECs exposed to PSS or LSS. H-I, Representative immunofluorescence images (**H**) and quantification (**I**) of KLF4 fluorescence in HUVECs exposed to physiological shear stress (PSS) or low shear stress (LSS). (**J**,**K**), Representative immunofluorescence images (**J**) and quantification (**K**) of LDs in HUVECs overexpressing KLF4 (oe-KLF4) or vector control under LSS. Quantification in (**K**) represents the relative fluorescence intensity of LDs. LSS, low shear stress; PSS, physiological shear stress; KLF4, Krüppel-like factor 4. Results are shown as mean ± SEM, with comparisons between two groups conducted using an unpaired two-tailed Student’s *t* test. *n* = 6 for both cell and animal experiments.

To elucidate the molecular mechanisms by which pathological mechanical forces induce endothelial lipid metabolism disorders, we performed a comprehensive bioinformatic screening by integrating three independent transcriptomic datasets: GSE45225 (LSS vs. PSS in HUVECs), GSE103672 (OSS vs. PSS in HUVECs), and GSE197366 (DF vs. SF in PCL mice model). Using stringent filtering criteria (|log2FC| > 1 and *p* < 0.05), we identified differentially expressed genes (DEGs) across these studies and intersected them with a curated LDs-related gene set retrieved from the GeneCards database. A four-way Venn diagram analysis revealed that KLF4 was the central candidate gene consistently associated with LD regulation across all in vitro and in vivo pathological flow models (Figure 3A). Subsequent visualization of DEGs across the three datasets demonstrated a consistent and significant downregulation of KLF4 in response to pathological SS (Figure 3B–D). Given KLF4 was established as a pivotal transcriptional regulator in endothelial mechano-transduction and its robust correlation with LD dynamics in our multi-dimensional screening, it was selected as the primary target for further functional investigation [18]. RT-qPCR analysis revealed that KLF4 mRNA expression was significantly reduced in the AA compared with the TA of HFD-fed ApoE^−/−^ mice (Figure 3E). Consistently, Western blot analysis confirmed a marked decrease in KLF4 protein levels in the AA region relative to the TA region (Figure 3F and Figure S3A). Parallel to the results observed in the aortic segments, the PCL model further validated that KLF4 expression is markedly suppressed in response to LSS (Figure S3B–E). In vitro, exposure of HUVECs to LSS resulted in a pronounced reduction in KLF4 expression compared with PSS, as demonstrated by immunofluorescence staining (Figure 3H,I) and Western blot analysis (Figure 3G and Figure S3F). To determine whether KLF4 modulates LD accumulation under LSS, HUVECs were transduced with adenovirus to overexpress KLF4. Western blot analysis confirmed effective overexpression, with significantly elevated KLF4 levels in the oe-KLF4 + LSS group compared with the Vector + LSS group (Figure S3G,H). Immunofluorescence analysis further revealed that KLF4 overexpression markedly attenuated lipid droplet accumulation under LSS conditions (Figure 3J,K). In addition, to investigate whether KLF4 regulates lysosome-dependent lipophagy, Western blot analysis showed that KLF4 overexpression did not alter LAMP1 expression but significantly reduced p62 levels and the LC3B-II/I ratio under LSS conditions (Figure S3I–K), indicating reversed lipophagy. Moreover, immunofluorescence staining demonstrated that KLF4 overexpression restored lysosomal acidification, as evidenced by increased LysoTracker fluorescence intensity, without affecting LAMP1 expression (Figure S3L,M). Collectively, these findings indicate that KLF4 may function as a key mechanosensitive regulator linking mechanical stress to LD accumulation and lysosome-dependent lipophagy dysfunction in endothelial cells.

### 3.4. KLF4 Promotes TFEB Activation and Selectively Enhances Lysosomal Functional Competence Under Low Shear Stress

**Figure 4:**
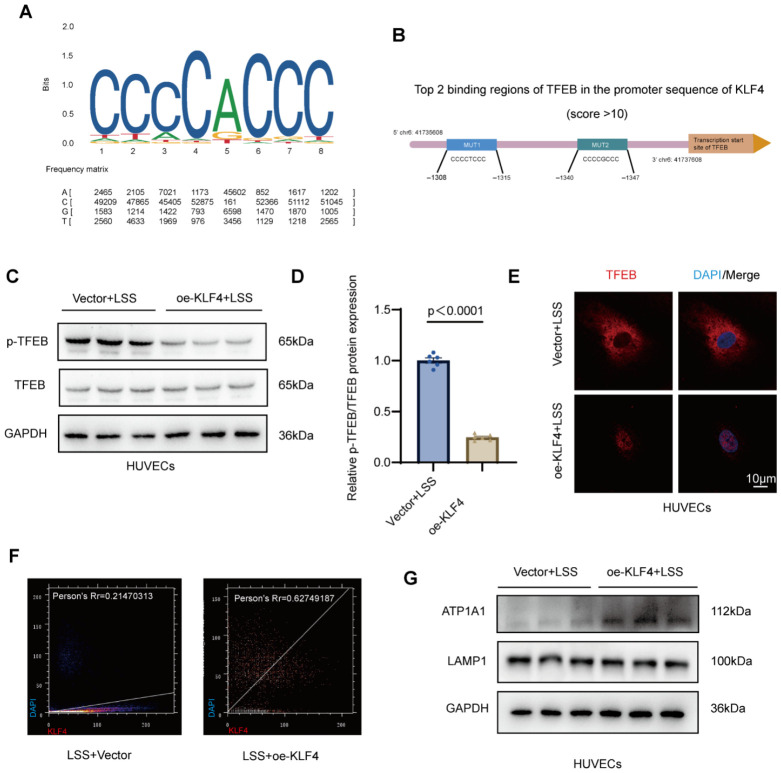
KLF4 promotes TFEB activation and selectively enhances lysosomal functional competence under low shear stress. (**A**), Motif analysis showing the conserved KLF4-binding sequence within the KLF4 promoter region. The motif sequence logo illustrates the conserved base preferences, where the total height of each stack indicates the sequence conservation at that position, and the relative height of each nucleotide letter represents its frequency. Colors represent different bases (A, T, C, and G) according to the standard color scheme. (**B**), Predicted top two TFEB-binding regions within the KLF4 promoter, indicating potential regulatory crosstalk. (**C**), Representative Western blot images of phosphorylated TFEB (p-TFEB) and total TFEB in HUVECs transduced with Vector or oe-KLF4 adenovirus and then subjected to LSS. (**D**), Quantification of p-TFEB/TFEB ratio based on analysis of Western blot bands. (**E**), Representative immunofluorescence images of TFEB (red) and nuclei (DAPI, blue) in HUVECs transduced with Vector or oe-KLF4 adenovirus under LSS. (**F**), Quantitative colocalization analysis of TFEB and DAPI signals, presented as Pearson’s correlation coefficient (Rr). KLF4 (red) and nuclei (DAPI, blue). (**G**), Western blot images of lysosomal markers ATP1A1 and LAMP1 in HUVECs transduced with Vector or oe-KLF4 under LSS. HUVECs, human umbilical vein endothelial cells; LSS, low shear stress; oe-KLF4, adenovirus-mediated KLF4 overexpression; TFEB, transcription factor EB; p-TFEB, phosphorylated TFEB; ATP1A1, Na^+^/K^+^-ATPase α1 subunit; LAMP1, lysosomal-associated membrane protein 1; DAPI, 4′,6-diamidino-2-phenylindole. Data are presented as mean ± SEM. Statistical analyses were performed using unpaired two-tailed Student’s *t* test, *n* = 6 independent experiments.

Given that KLF4 overexpression restored lysosomal acidification and attenuated LD accumulation under LSS, we next sought to elucidate the molecular mechanism underlying this lysosome-dependent effect. TFEB is widely recognized as a key transcriptional regulator of lysosomal biogenesis and autophagy-related gene expression, whose activity is primarily governed by its phosphorylation status and subcellular localization [19]. In its phosphorylated form, TFEB is retained in the cytoplasm, whereas dephosphorylation promotes its nuclear translocation and transcriptional activation of lysosomal genes [20,21]. Based on this regulatory paradigm, we examined whether KLF4 modulates TFEB activation under LSS conditions. Motif analysis identified a conserved KLF4-binding sequence (Figure 4A). In addition, bioinformatic prediction revealed two putative TFEB-binding regions within the promoter sequence of KLF4 (Figure 4B), suggesting a potential regulatory association between KLF4 and TFEB. However, to determine the functional directionality under LSS, we focused on whether KLF4 regulates TFEB activation. HUVECs were transduced with adenovirus to overexpress KLF4 under LSS conditions. Western blot analysis demonstrated that KLF4 overexpression markedly reduced phosphorylated TFEB levels compared with the Vector + LSS group, whereas total TFEB expression remained unchanged (Figure 4C,D). In addition, IF staining revealed pronounced nuclear translocation of TFEB in the oe-KLF4 + LSS group compared with Vector + LSS (Figure 4E). Quantitative colocalization analysis further demonstrated a substantial increase in the Pearson’s correlation coefficient between TFEB and DAPI signals following KLF4 overexpression (person’s Rr = 0.62749187) relative to the Vector + LSS group (person’s Rr = 0.21470313) (Figure 4F), confirming enhanced TFEB nuclear localization. To evaluate downstream lysosomal effectors, we next assessed ATP1A1 and LAMP1 expression. Western blot analysis showed that KLF4 overexpression significantly upregulated ATP1A1 expression under LSS, whereas LAMP1 levels remained unchanged (Figure 4G and Figure S4A,B). Taken together, these results demonstrate that KLF4 promotes TFEB dephosphorylation and nuclear translocation under LSS, thereby restoring lysosomal functional competence and contributing to the maintenance of endothelial lipid metabolic homeostasis.

### 3.5. Rapamycin Restores the KLF4/TFEB/ATP1A1 Axis to Attenuate LD Accumulation Driven by LSS In Vivo

**Figure 5:**
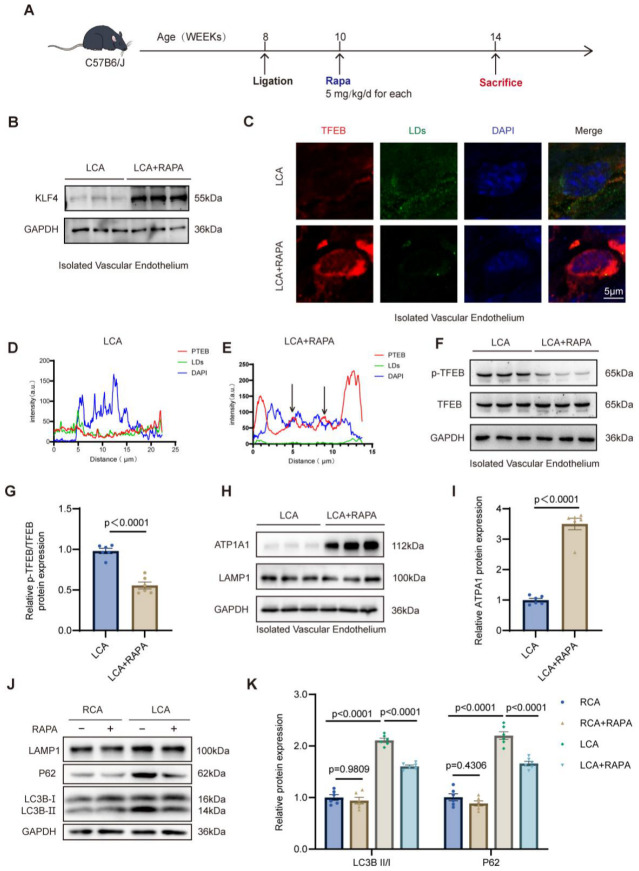
Rapamycin-mediated modulation of the KLF4/TFEB/lysosome axis in partial carotid ligation mice. (**A**), Schematic illustration of the in vivo experimental design. C57BL/6J mice underwent partial carotid ligation and received daily intraperitoneal injections of rapamycin (5 mg/kg) or an equal volume of saline for four weeks. (**B**), Representative Western blot images of KLF4 protein expression in LCA and LCA + RAPA groups. (**C**), Enface immunofluorescence images of LCA and LCA + RAPA groups, with TFEB (red), lipid droplets (LDs, green), and nuclei (DAPI, blue). (**D**), Fluorescence intensity line profile analysis (line scan) of TFEB and DAPI in LCA group. (**E**), Fluorescence intensity line profile analysis (line scan) of TFEB and DAPI in LCA + RAPA group. The arrows indicate the co-localization of the fluorescence channels. (**F**), Representative Western blot images of p-TFEB and total TFEB in LCA and LCA + RAPA groups. (**G**), Densitometric quantification of the p-TFEB/TFEB ratio in LCA and LCA + RAPA groups. (**H**), Representative Western blot images of LAMP1 and ATP1A1 in LCA and LCA + RAPA groups. (**I**), Densitometric quantification of ATP1A1 in LCA and LCA + RAPA groups. (**J**), Representative Western blot images of LAMP1, LC3B, and P62 in RCA, RCA + RAPA, LCA, and LCA + RAPA groups. (**K**), Densitometric quantification of LAMP1, LC3B-II/I ratio, and P62 in RCA, RCA + RAPA, LCA, and LCA + RAPA groups. LCA, ligated left carotid artery; RCA, right carotid artery; RAPA, rapamycin; LD, lipid droplet; ATP1A1, Na^+^/K^+^-ATPase α1 subunit; LAMP1, lysosomal-associated membrane protein 1; P62, sequestosome-1; LC3B, microtubule-associated protein 1 light chain 3B. Statistical analyses were performed using unpaired two-tailed Student’s *t* test or two-way ANOVA followed by Tukey’s multiple comparison test. *n* = 6 mice per group.

Considering the regulatory role of the KLF4/TFEB axis in lysosome-dependent lipophagy, we examined whether autophagy induction by rapamycin could alleviate LSS-induced LD accumulation in vivo. C57BL/6J mice undergoing partial carotid ligation received daily intraperitoneal injections of RAPA (5 mg/kg) or an equivalent volume of saline for four weeks (Figure 5A) [22]. Following treatment, the LCA + RAPA group exhibited a notable reduction in endothelial LD content relative to the LCA group (Figure S5A,B). In parallel, Western blot analysis demonstrated that RAPA treatment significantly upregulated KLF4 protein expression within the ligated LCA relative to the vehicle-treated LCA group (Figure 5B and Figure S5C). To evaluate TFEB activation, we performed enface immunofluorescence staining, which revealed that RAPA treatment markedly enhanced the nuclear translocation of TFEB and concurrently reduced LD burden in the LCA (Figure 5C). Notably, fluorescence intensity line profile analysis (line scan) confirmed minimal overlap between TFEB and DAPI signals in untreated LCA, whereas RAPA treatment yielded a robust increase in nuclear TFEB occupancy (Figure 5D,E). Consistent with these findings, RAPA treatment significantly suppressed TFEB phosphorylation, as evidenced by a decreased p-TFEB/TFEB ratio in the LCA, indicating a shift toward its transcriptionally active state (Figure 5F,G). We further examined downstream lysosomal effectors and observed that RAPA-mediated TFEB activation was accompanied by a marked increase in ATP1A1 expression, while LAMP1 levels remained stable (Figure 5H,I and Figure S5G). Enface imaging utilizing LysoTracker and LAMP1 further corroborated that RAPA successfully restored lysosomal acidification without altering total lysosomal content (Figure S5D–F). To verify the restoration of lipophagy, we assessed the LC3B-II/I ratio and P62 levels across experimental groups. In the LCA, RAPA effectively reversed the LSS-induced accumulation of P62 and the elevation of the LC3B-II/I ratio, while LAMP1 expression remained constant (Figure 5J,K and Figure S5H). These data support the hypothesis that RAPA mitigates LSS-induced lipidosis by augmenting KLF4 expression, promoting TFEB nuclear recruitment, and enhancing ATP1A1-dependent lysosomal acidification, thereby restoring lysosome-dependent lipophagy and maintaining lipid metabolic homeostasis.

### 3.6. Rapamycin Alleviates LSS-Driven Endothelial Lipidosis Through Restoration of the KLF4/TFEB/ATP1A1 Axis In Vitro

**Figure 6:**
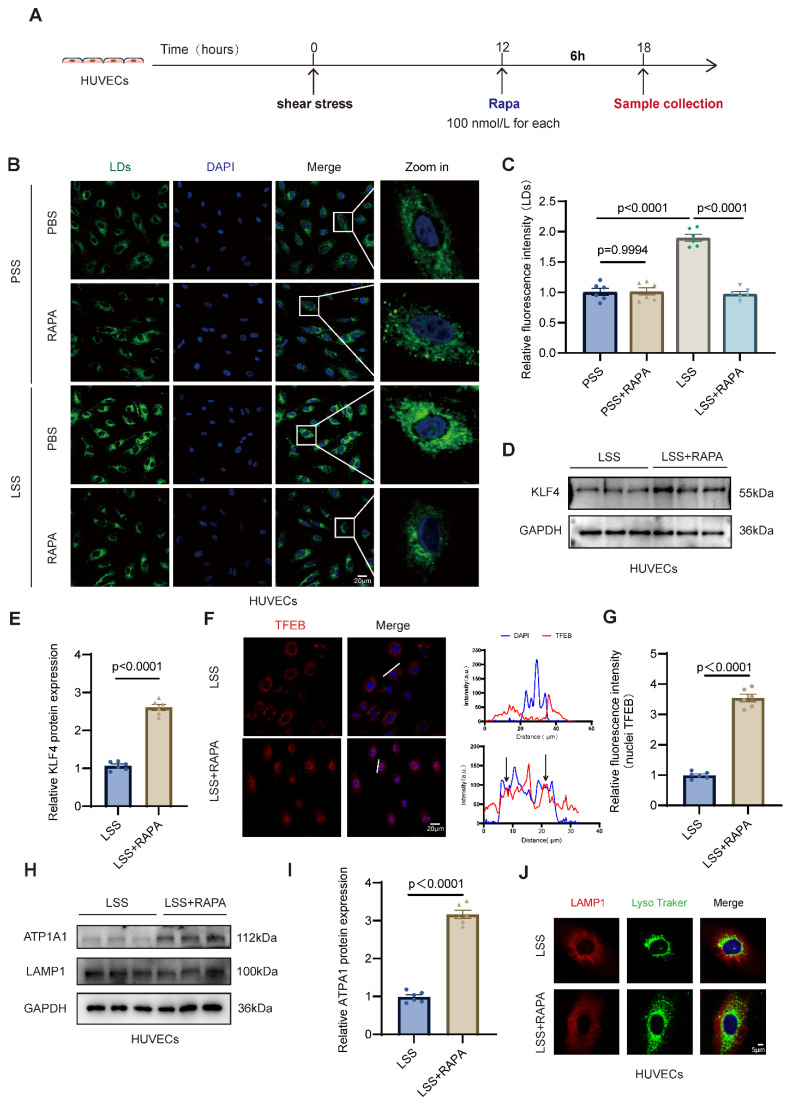
The protective effect of rapamycin on LSS-induced endothelial lipid dysregulation is mediated by the KLF4/TFEB axis. (**A**), Schematic illustration of the in vitro experimental design. HUVECs were exposed to 12 h of low shear stress (LSS) or physiological shear stress (PSS), followed by treatment with PBS or rapamycin for an additional 6 h before sample collection. (**B**), Representative immunofluorescence images of lipid droplets (LDs, green) and nuclei (DAPI, blue).in PSS + PBS, PSS + RAPA, LSS + PBS, and LSS + RAPA groups. (**C**), Quantification of LD fluorescence intensity in the four experimental groups of HUVECs. (**D**), Representative Western blot images of KLF4 protein expression in LSS and LSS + RAPA groups. (**E**), Densitometric quantification of KLF4 protein expression in LSS and LSS + RAPA groups. (**F**), Representative immunofluorescence images of TFEB (red) and nuclei (DAPI, blue) in LSS and LSS + RAPA groups, along with corresponding fluorescence intensity line profile analyses. The white lines delineate the cells selected for the visualization of fluorescence intensity, and the arrows indicate the co-localization of the fluorescence channels. (**G**), Quantification of nuclear TFEB fluorescence intensity in LSS and LSS + RAPA groups. (**H**), Representative Western blot images of ATP1A1 and LAMP1 protein expression in LSS and LSS + RAPA groups. (**I**), Densitometric quantification of ATP1A1 and LAMP1 protein expression in LSS and LSS + RAPA groups. (**J**), Representative immunofluorescence images of LAMP1 (red), LysoTracker (green), and nuclei (DAPI, blue) in LSS and LSS + RAPA groups. HUVECs, human umbilical vein endothelial cells; LSS, low shear stress; PSS, physiological shear stress; RAPA, rapamycin; LD, lipid droplet; ATP1A1, Na^+^/K^+^-ATPase α1 subunit; LAMP1, lysosomal-associated membrane protein 1. Statistical analyses were performed using unpaired two-tailed Student’s *t* test or two-way ANOVA followed by Tukey’s multiple comparison test. *n* = 6 independent experiments.

To further determine whether RAPA exerts direct protective effects on endothelial cells exposed to LSS, HUVECs were subjected to 12 h of LSS or PSS, followed by PBS or rapamycin treatment for an additional 6 h (Figure 6A). Immunofluorescence staining demonstrated that LSS markedly increased LD accumulation compared with PSS, whereas RAPA treatment significantly reduced LD content under LSS conditions (Figure 6B,C), indicating that pharmacological autophagy induction alleviates LSS-induced lipid deposition in vitro. We next examined whether this protective effect involves modulation of the KLF4/TFEB signaling axis. Western blot analysis demonstrated that RAPA significantly upregulated KLF4 protein expression in HUVECs exposed to LSS (Figure 6D,E), suggesting that the KLF4/TFEB axis might be a key mediator of RAPA-conferred endothelial protection. Since TFEB transcriptional activity is primarily governed by its subcellular partitioning, we next characterized its spatial distribution via immunofluorescence staining. Under LSS conditions, TFEB was sequestered predominantly within the cytoplasm, showing minimal colocalization with DAPI-stained nuclei. Conversely, RAPA treatment markedly promoted the nuclear recruitment of TFEB. This shift was rigorously confirmed by fluorescence intensity line profile analysis and quantitative determination of nuclear TFEB signals (Figure 6F,G), indicating that RAPA facilitates TFEB activation by driving its nuclear translocation under hemodynamic stress. These findings indicate that RAPA re-establishes the KLF4/TFEB axis by driving TFEB nuclear translocation, thereby counteracting LSS-induced pathological lipid deposition. Likewise, to evaluate downstream lysosomal effectors, we measured ATP1A1 and LAMP1 expression. Western blot analysis revealed that RAPA significantly upregulated ATP1A1 protein levels, whereas LAMP1 expression remained unchanged (Figure 6H,I and Figure S6C). Consistently, immunofluorescence staining using LysoTracker and LAMP1 demonstrated enhanced lysosomal acidification in rapamycin-treated cells without alterations in total lysosomal content (Figure 6J and Figure S6D), indicating selective restoration of lysosomal functional competence. Finally, we assessed lipophagic flux by examining P62 and LC3B levels. Western blot analysis showed that rapamycin significantly reduced P62 accumulation and normalized the LC3B-II/I ratio under LSS conditions (Figure S6A,B), suggesting improved lipophagy. Collectively, these results demonstrate that in HUVECs exposed to LSS, RAPA suppresses LD accumulation by activating the KLF4/TFEB signaling axis, enhancing ATP1A1-dependent lysosomal acidification, and restoring lysosome-mediated lipophagy.

### 3.7. Rapamycin Attenuates Site-Specific Atherosclerotic Progression Through Restoration of the KLF4/TFEB/ATP1A1 Axis in ApoE^−/−^ Mice

**Figure 7:**
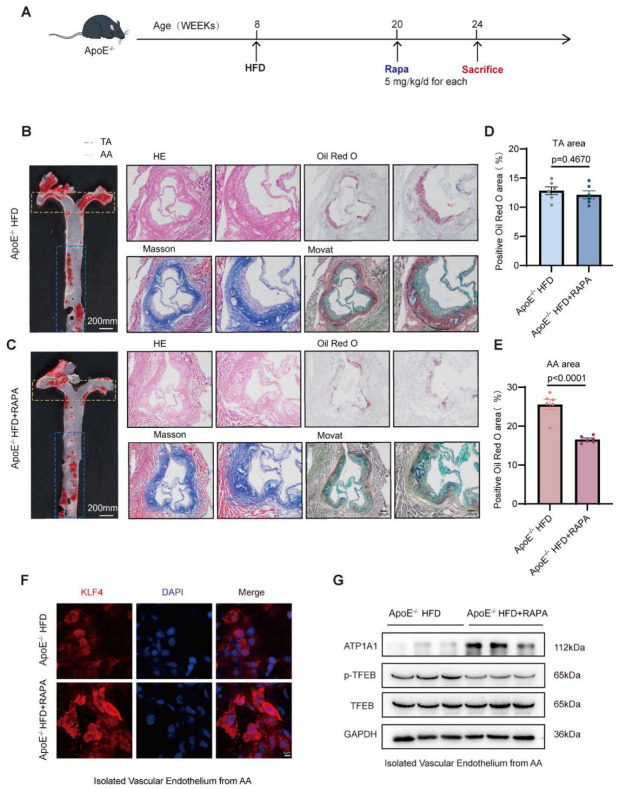
Rapamycin modulates KLF4/TFEB/ATP1A1 axis and plaque composition in ApoE^−/−^ HFD mice. (**A**), Schematic diagram of the experimental design. Eight-week-old ApoE^−/−^ mice were fed a high-fat diet (HFD) and subsequently treated with rapamycin for four weeks prior to tissue harvest. (**B**,**C**), Representative enface Oil Red O and HE/Oil Red O/Masson/staining images of aorta and aortic root from ApoE^−/−^ HFD and ApoE^−/−^ HFD + RAPA groups. (**D**), Quantification of Oil Red O positive plaque area in TA regions. (**E**), Quantification of Oil Red O positive plaque area in AA regions. (**F**), Enface immunofluorescence images of AA regions from ApoE^−/−^ HFD and ApoE^−/−^ HFD + RAPA groups, showing KLF4 (red) and nuclei (DAPI, blue). (**G**), Representative Western blot images of ATP1A1, p-TFEB, and TFEB in AA regions from ApoE^−/−^ HFD and ApoE^−/−^ HFD + RAPA groups. RAPA, rapamycin; AA, aortic arch; TA, thoracic aorta; DAPI, 4′,6-diamidino-2-phenylindole; ATP1A1, Na^+^/K^+^-ATPase α1 subunit. Statistical analyses were performed using unpaired two-tailed Student’s *t* test. *n* = 6 independent experiments.

To evaluate the atheroprotective effects of RAPA-mediated KLF4/TFEB activation, eight-week-old ApoE^−/−^ mice were fed a HFD and administered RAPA for four weeks (Figure 7A). Oil Red O staining of the whole aorta revealed a significant reduction in lipid deposition in RAPA-treated mice compared with ApoE^−/−^ HFD controls (Figure 7B,C and Figure S7A). Intriguingly, this reduction was predominantly observed in AA regions, whereas plaque burden in the TA showed no significant difference between the two groups (Figure 7D,E). These data suggest that the protective effect of RAPA is more pronounced in vascular regions exposed to LSS. To further assess plaque composition and stability, aortic root sections were analyzed via Oil Red O, H&E, Masson’s trichrome, and MOVAT pentachrome staining (Figure 7B,C). Oil Red O and H&E staining demonstrated reduced plaque area in the ApoE^−/−^ HFD + RAPA group (Figure S7B,C). Moreover, Masson staining revealed increased collagen content within aortic root lesions in RAPA-treated mice (Figure S7D), suggesting structural remodeling of the fibrous component and potential enhancement of plaque stability. These results collectively demonstrate that RAPA not only reduces lipid deposition but also promotes features associated with plaque stabilization. Building upon the mechanistic link between RAPA and the KLF4/TFEB axis identified in vitro and in vivo, we next examined whether this signaling cascade is similarly activated in the atherosclerotic vasculature. Enface immunofluorescence staining of the AA showed a marked KLF4 intensity increase in RAPA-treated mice compared with ApoE^−/−^ HFD controls (Figure 7F and Figure S7C). Consistently, Western blot analysis of AA tissue lysates revealed upregulation of ATP1A1 protein expression accompanied by a significant reduction in the p-TFEB/TFEB ratio in the ApoE^−/−^ HFD + RAPA group (Figure 7G and Figure S7E), indicating enhanced TFEB activation and downstream lysosomal effector engagement in vivo. Importantly, unlike previous studies that primarily focus on systemic plaque burden, our findings highlight a hemodynamics-dependent protective effect of RAPA [23]. Specifically, restoration of the KLF4/TFEB/ATP1A1 axis appears to relieve LSS-induced lysosomal dysfunction, thereby enhancing autophagy-dependent lipid clearance and attenuating local plaque progression.

## 4. Discussion

The regulatory interplay between biomechanical forces and endothelial metabolism has been increasingly recognized as a cornerstone of vascular homeostasis. Although previous studies have established the roles of SS in modulating endothelial inflammation and oxidative stress, the precise mechanisms by which local hemodynamic regimes dictate endothelial lipid metabolic dysregulation remain poorly defined. In this investigation, we demonstrate that LSS acts as a primary driver of endothelial lipid dysregulation by triggering the pathological accumulation of LDs. By deconstructing the KLF4/TFEB/ATP1A1 signaling axis, we show that LSS impairs lysosome-dependent lipophagy through the suppression of TFEB nuclear translocation and the disruption of ATP1A1-mediated lysosomal acidification. Crucially, our findings reveal that Rapamycin restores this mechanometabolic bridge, thereby re-establishing lipophagic flux and selectively attenuating plaque progression in LSS-prone regions. These results provide a novel perspective on the EC-specific protective effects of the KLF4/TFEB/ATP1A1 axis and identify endothelial LD homeostasis as a promising therapeutic target for the precision treatment of AS.

AS is well recognized as a disease with strong spatial predilection, preferentially developing at arterial bifurcations and curvatures exposed to disturbed flow and LSS [24]. Although previous studies have established that LSS promotes endothelial inflammation, oxidative stress, and phenotypic activation, its direct impact on endothelial lipid metabolic homeostasis has been less well defined [25,26,27]. As specialized organelles, LDs serve not only as metabolic reservoirs but also as pivotal regulators of intracellular lipid homeostasis [28,29]. Under physiological conditions, endothelial LDs undergo dynamic regulation through a balance of biosynthesis and lipolysis to maintain metabolic equilibrium [30]. Pathological LD accumulation has been implicated in a broad spectrum of cardiometabolic and degenerative disorders [31,32]. This study extends these paradigms by demonstrating that LSS is a critical biomechanical trigger of endothelial LD deposition, thereby linking disturbed flow to lipid metabolic dysfunction. Our findings align with those of Boutagy et al. [33], who reported that endothelial ATGL deficiency induces LD accumulation and triggers a cascade of ER stress and NF-κB activation, ultimately driving endothelial dysfunction and atherosclerotic progression. Correspondingly, our data reveal that LSS similarly modulates key metabolic enzymes, leading to a comparable state of lipid imbalance. Mechanistically, this LSS-induced sequestration is primarily driven by defective lipophagic flux. Increased P62 level and altered LC3B-II/I ratio suggest impaired degradation rather than increased lipid synthesis alone [34,35]. As the irreplaceable degradative hub, lysosomes require a highly acidic environment to maintain the optimal catalytic activity of their resident hydrolases. These enzymes exhibit optimal catalytic activity exclusively within a low-pH environment; thus, lysosomal acidification is fundamental for maintaining physiological autophagic flux. Indeed, impaired acidification has been identified as a core mechanism underlying autophagic stagnation [36]. Given that autophagic degradation depends critically on lysosomal competence, we observed that lysosomal acidification is significantly reduced under LSS, whereas LAMP1 expression remains unchanged. This dissociation suggests that lysosomal functional competence, rather than lysosome abundance, is compromised under LSS. These findings are consistent with prior evidence that lysosomal dysfunction plays a central role in atherogenesis [37]. Accordingly, this study found that LSS disrupts endothelial lipid clearance primarily through impairment of lysosome-dependent lipophagy.

KLF4 has been widely reported to maintain endothelial quiescence and exert anti-inflammatory effects in vascular biology [38,39]. Based on public GEO dataset analysis and bioinformatic screening, our study identify KLF4 as a critical mechanosensitive factor linking LSS to LD pathological deposition. In the context of vascular biology, the protective role of KLF4 is well-established; previous studies have demonstrated that KLF4 can suppress senescence-associated secretory phenotype (SASP) in oxidized low-density lipoprotein ox-LDL-treated endothelial cells via the PDGFRA/NAMPT/mitochondrial ROS pathway [40]. However, its role in regulating lysosomal acidification and lipophagy has not been clearly defined. In our study, we show that LSS suppresses KLF4 expression. TFEB, a master regulator of lysosomal biogenesis, was largely retained in the cytoplasm with increased phosphorylation, indicating reduced nuclear translocation and transcriptional activity [41]. It has been well established that the phosphorylation of TFEB promotes its cytosolic retention through 14-3-3 binding, whereas dephosphorylation facilitates its nuclear translocation and activation of lysosomal and autophagy genes [42]. In our research, we found that restoration of KLF4 expression rescues lysosomal acidification and promotes activation of TFEB, thereby effectively restoring lysosomal acidification and reactivating downstream lipophagy under LSS. Maintenance of lysosomal acidity depends critically on coordinated ion transport and proton gradients across the lysosomal membrane [43]. While previous studies have linked ion homeostasis to lysosomal function, the role of ATP1A1 in endothelial lipophagy under shear stress conditions has not been previously characterized. Our findings suggest that ATP1A1 participates in preserving lysosomal acidification and thereby sustaining lipophagic flux. Together, these results delineate a mechanistic axis in which LSS suppresses KLF4, leading to impaired TFEB activation, disruption of ATP1A1-associated lysosomal acidification, defective lipophagy, and ultimately pathological lipid droplet accumulation in endothelial cells. Conversely, overexpression of KLF4 suppressed TFEB phosphorylation, thereby facilitating its nuclear translocation and restoring lysosomal biogenesis, significantly attenuating pathological lipid droplet accumulation in endothelial cells exposed to LSS.

Notably, RAPA, a classic mTOR inhibitor, has been shown to effectively restore impaired autophagic flux and exert protective effects across diverse pathological contexts [44]. In the realm of AS therapy, autophagy restoration emerges as a pivotal pathway for maintaining intracellular lipid homeostasis, whereby moderate autophagic activation facilitates LD degradation and curbs foam cell formation. Our findings suggest that the reduction in atherosclerotic lesion area observed with RAPA treatment reflects a synergistic integration of its systemic anti-inflammatory actions and the restoration of endothelial lipid metabolism. While RAPA is a potent inhibitor recognized for its capacity to suppress the accumulation of inflammatory cells and the deposition of extracellular matrix proteins [45,46], our investigation highlights a unique and novel mechanistic facet involving the regulation of the endothelial KLF4/TFEB/ATP1A1 axis. Consistent with this, recent studies demonstrate that Vismodegib, a Hedgehog pathway small-molecule inhibitor, activates autophagy to accelerate cholesterol efflux, thereby reducing lipid deposition in early-stage AS [47]. As the primary “sentinels” of the vascular system, endothelial cells subjected to LSS function as a primary “pro-inflammatory trigger” [48]. Our data demonstrate that RAPA reverses LSS-induced impairment of the KLF4/TFEB/ATP1A1 signaling axis and reduces pathological lipid droplet accumulation, thereby ameliorating lysosomal dysfunction. This reversal creates the necessary conditions for the subsequent reduction in the recruitment of blood-derived inflammatory cells. By restoring this axis, RAPA treatment rebuilds endothelial “metabolic fitness,” serving as a functional barrier that limits subsequent inflammatory cell infiltration. These results elucidate that RAPA-mediated metabolic improvement functions as an upstream event to attenuate the local inflammatory milieu, providing a more holistic perspective on the molecular mechanisms underlying plaque regression. Consequently, while the classical systemic anti-inflammatory effects of RAPA are well-established, our data reveal that its involvement in endothelial-specific metabolic regulation represents a novel mechanistic dimension contributing significantly to the overall therapeutic outcome.

There are several limitations to this study. First, while our findings establish KLF4 as a regulator of TFEB phosphorylation, the precise mechanisms regarding specific kinases, phosphatases, or KLF4-mediated chromatin remodeling remain to be fully elucidated. Second, while RAPA effectively restores the KLF4/TFEB/ATP1A1 axis and lysosomal function in endothelial cells, the long-term effects of such pharmacological intervention on plaque progression and stability in vivo require further evaluation. Third, this study primarily focuses on endothelial cells; the contributions of other vascular cell types, including smooth muscle cells and macrophages, to LSS-dependent lipid dysregulation remain to be elucidated. Last, although we employed HUVECs as an in vitro model to explore the molecular mechanisms of endothelial dysfunction, our experimental findings remained remarkably consistent across both the cellular and mouse models. While HUVECs are a widely accepted model for studying mechano-transduction, the inherent differences between venous and arterial cells, as well as species variations, necessitate further exploration. Therefore, future research will focus on integrating a multi-tiered platform of cell models, including primary human aortic endothelial cells (HAECs), patient-derived induced pluripotent stem cell (iPSC)-derived ECs, and advanced 3D organ-on-a-chip systems. This comprehensive multi-model validation strategy will be instrumental to substantiate our current findings and effectively bridge the gap between in vitro experimental data and in vivo arterial pathology.

## 5. Conclusions

**Figure 8:**
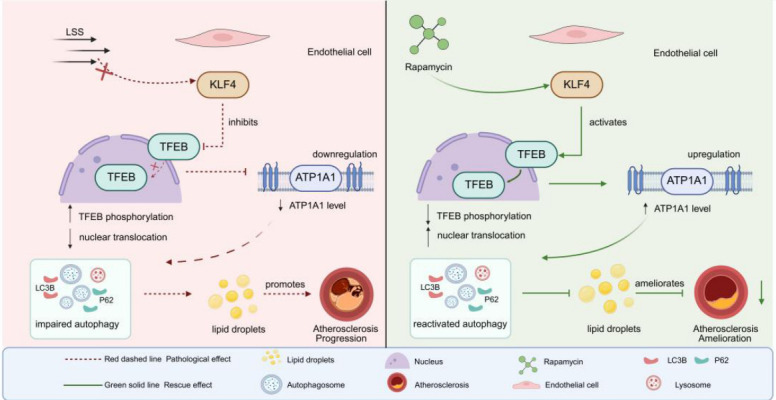
Schematic illustration. Low shear stress drives endothelial lipid accumulation and impairs lysosomal lipophagy by suppressing the KLF4/TFEB/ATP1A1 axis, thereby promoting site-specific atherosclerosis in LSS regions. Notably, reactivating this axis with Rapamycin restores lysosomal function, enhances lipid clearance, and attenuates plaque burden. Created in BioRender. Chen, P. (2026). https://www.biorender.com/xuh9gwr (accessed on 24 February 2026).

In conclusion, our study reveals that LSS drives endothelial lipid accumulation and impairs lysosomal lipophagy by suppressing the KLF4/TFEB/ATP1A1 axis, thereby promoting site-specific AS in LSS regions. Notably, reactivating this axis with Rapamycin restores lysosomal function, enhances lipid clearance, and improves plaque stability (Figure 8). These findings highlight targeting endothelial KLF4/TFEB-mediated lysosomal pathways as a potential strategy to combat AS in hemodynamically vulnerable regions.

## Data Availability

The original contributions presented in the study are included in the article and Supplementary Materials, further inquiries can be directed to the corresponding authors.

## Supplementary Materials

The following supporting information can be downloaded at: https://www.mdpi.com/article/10.3390/jcdd13050213/s1. References [13,17,22,23,37,49,50,51,52,53] are cited in the Supplementary Materials.
