# Supplementary material for: Low Shear Stress Promotes Atherosclerosis by Mediating Pathological Accumulation of Endothelial Lipid Droplets via the KLF4/TFEB/ATP1A1 Axis
Source: J Cardiovasc Dev Dis. 2026 May 15;13(5):213. doi: 10.3390/jcdd13050213 (PMC13207403; doi:10.3390/jcdd13050213)
Supplement: Supplementary file 1 [file jcdd-13-00213-s001.zip › jcdd-4314498-supplementary_File_S1.pdf]

SUPPLEMENTARY INFORMATION

Low Shear Stress Promotes Atherosclerosis by Mediating Pathological Accumulation of Endothelial Lipid Droplets Via the KLF4/TFEB/ATP1A1 Axis

Content:

Figure S1-S7, Table S1

Supplementary figures

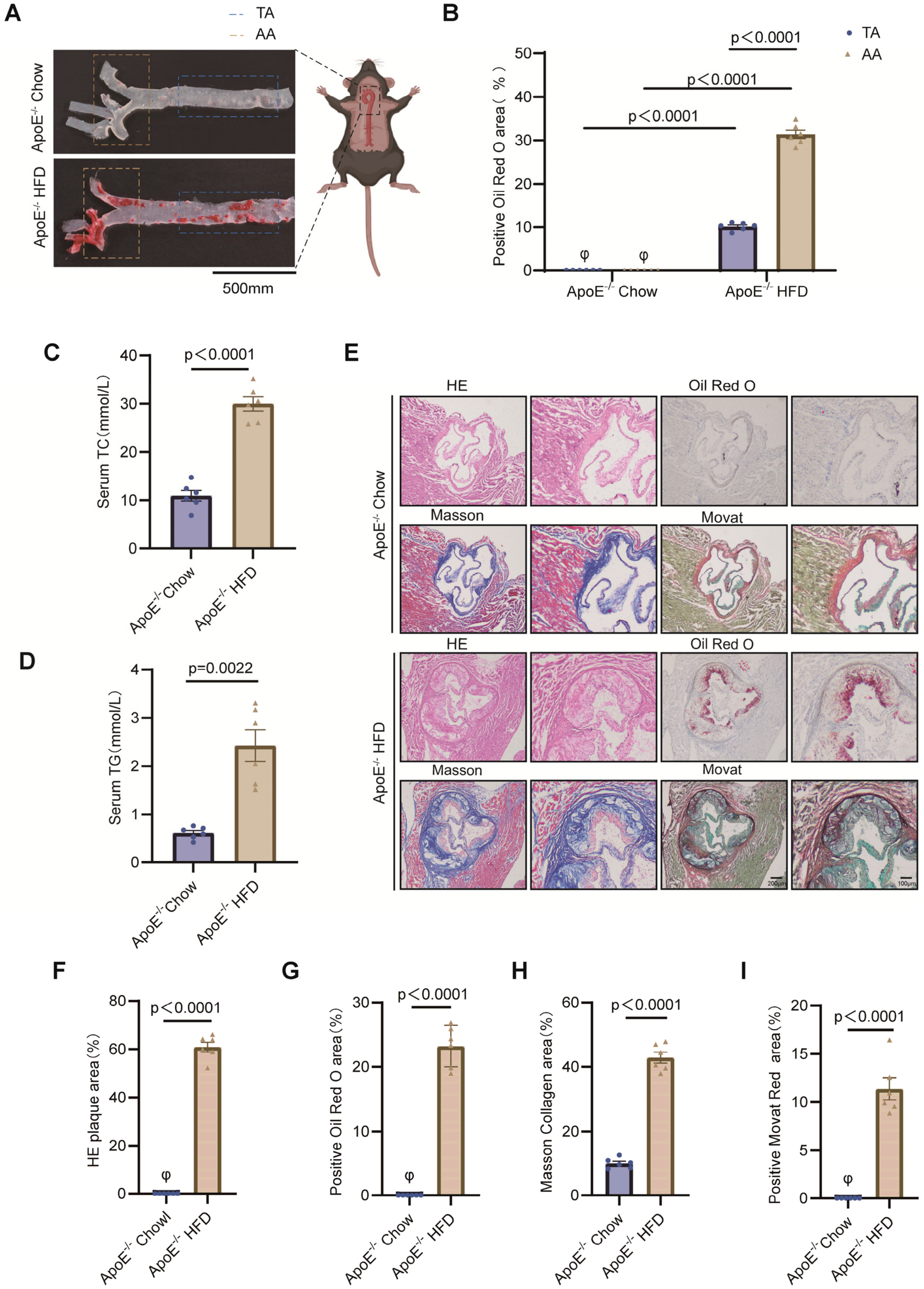

Figure S1: Aortic arch plaque distribution and aortic root pathological features in high-fat diet–fed ApoE⁻/⁻ mice.

A–B, Representative Oil Red O staining images of aortas from chow-fed control and high-fat diet–fed ApoE⁻/⁻ mice, with bar graphs showing quantitative comparison of

plaque area in the aortic arch (AA) and thoracic aorta (TA) regions. C–D, ELISA-based quantification of fasting serum triglyceride (TG) and total cholesterol (TC) levels in the two groups. E, Representative images of hematoxylin and eosin (HE), Oil Red O, Masson's trichrome, and Movat's pentachrome staining of the aortic root. High-magnification views of fixed fields are shown on the right. Scale bars: 200 μm (overview) and 100 μm (magnified images). F, Quantification of the ratio of plaque area to luminal area in the aortic root based on HE staining . G, Quantification of Oil Red O positive area relative to luminal area in the aortic root. H, Quantification of Masson's collagen positive area. I, Quantification of the ratio of Movat's acidic mucopolysaccharides–positive area to luminal area in the aortic root. φ indicates absence of plaque deposition. ApoE indicates apolipoprotein E; TC, total cholesterol; TG, triglyceride; HE, hematoxylin and eosin staining; Masson, Masson's trichrome staining; Movat, Movat's pentachrome staining; SEM, standard error of the mean. All data are presented as mean ± SEM. Statistical analyses were performed using one-sample t test or unpaired two-tailed Student's t test, as appropriate. n=6 mice per group.

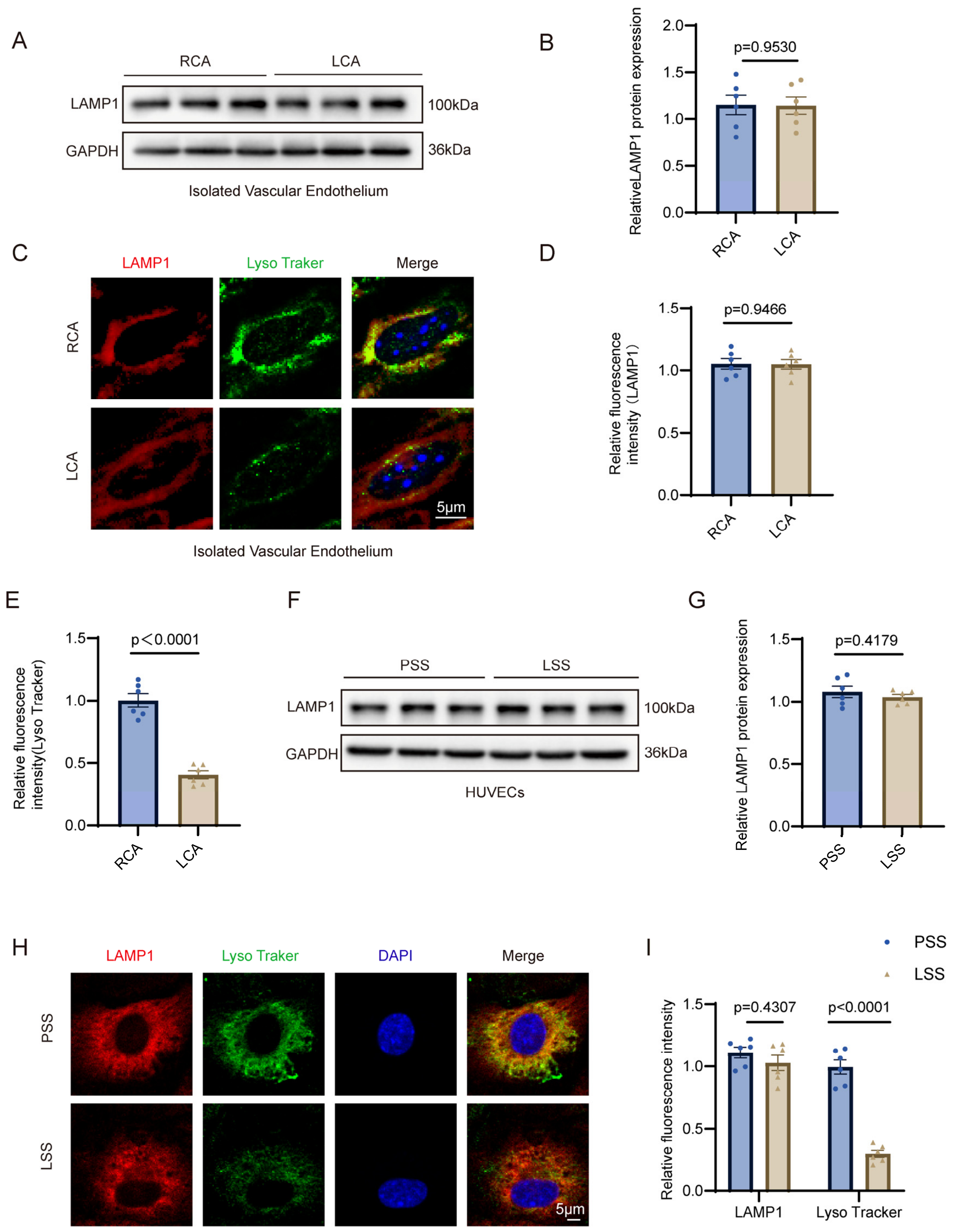

Figure S2 Impaired lysosomal acidification in LSS-mediated lipophagy.

A, Western blot analysis of LAMP1 levels in the right carotid artery (RCA) and left carotid artery (LCA).B, Quantification of LAMP1 protein expression.C, Representative enface immunofluorescence images of LAMP1 (red) and Lyso Tracker (green) in the endothelium of RCA and LCA. D-E, Quantification of LAMP1 and Lyso Tracker fluorescence intensity in RCA and LCA. F, Western blot analysis of LAMP1 levels in HUVECs exposed to low shear stress (LSS) or physiological shear stress (PSS).G, Quantification of LAMP1 protein expression. H, Representative

immunofluorescence images of LAMP1 (red) and LysoTracker (green) in HUVECs exposed to LSS or PSS. I, Quantification of LysoTracker fluorescence intensity in HUVECs. LCA, left carotid artery; RCA, right carotid artery; HUVEC, human umbilical vein endothelial cell; LSS, low shear stress; PSS, physiological shear stress; LAMP1, lysosomal-associated membrane protein 1; LysoTracker, a lysosomal marker; SEM, standard error of the mean. All data are presented as mean ± SEM. Statistical analyses were performed using unpaired two-tailed Student's t test or two-way ANOVA followed by Tukey's multiple comparison test. n = 6 for both cell and animal experiments.

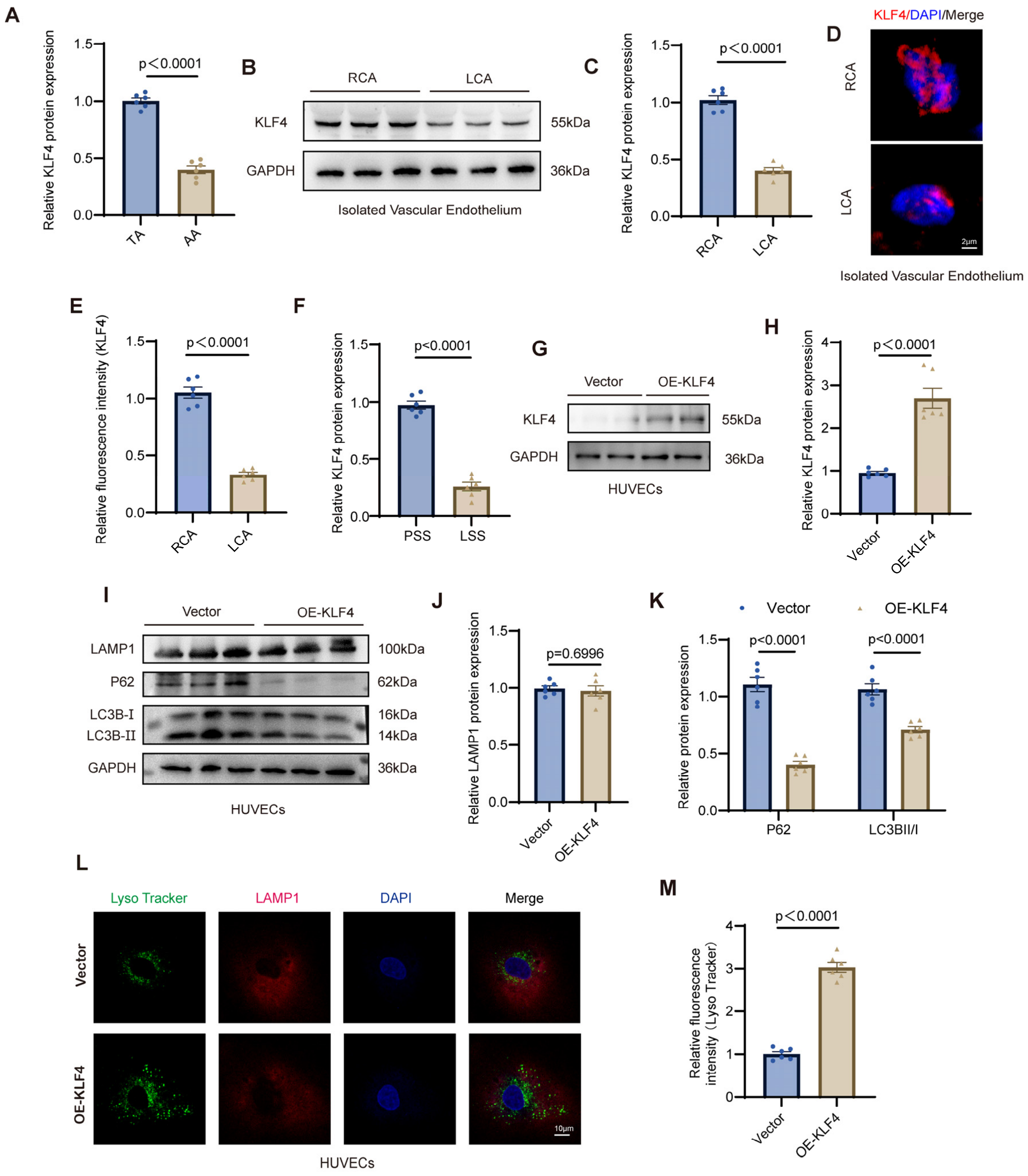

Figure S3 KLF4 overexpression restores lysosomal acidification and lipophagy under LSS.

A, Quantitative analysis of KLF4 protein expression in the TA and AA of HFD-fed ApoE$^{-/-}$ mice. B-C. Western blot and quantitative analysis of KLF4 protein expression in RCA and LCA regions of PCL mice. D-E,Enface immunofluorescence staining and quantitative analysis of KLF4 in the left carotid artery (LCA) and right carotid artery (RCA) after PCL. F, Western blot quantitative analysis of KLF4 protein expression in HUVECs exposed to PSS or LSS. G, Western blot analysis of KLF4 in HUVECs transduced with adenoviral-mediated KLF4 overexpression (oe-KLF4) or adenoviral vector control. H, Quantification of KLF4 protein expression from . I–K, Western blot

analysis (I) and quantification (J, K) of LAMP1, p62, and LC3B-II/I in HUVECs overexpressing KLF4 under LSS. L, Representative immunofluorescence images of LysoTracker in HUVECs overexpressing KLF4 under LSS. M, Quantification of LysoTracker fluorescence intensity from. LAMP1, lysosomal-associated membrane protein 1; p62, sequestosome-1; LC3B, microtubule-associated protein 1 light chain 3B; LysoTracker, lysosomal dye; SEM, standard error of the mean. All data are presented as mean ± SEM. Comparisons between groups were performed using unpaired two-tailed Student's t test or two-way ANOVA with Tukey's multiple comparison post hoc test. n = 6 for both cell and animal experiments.

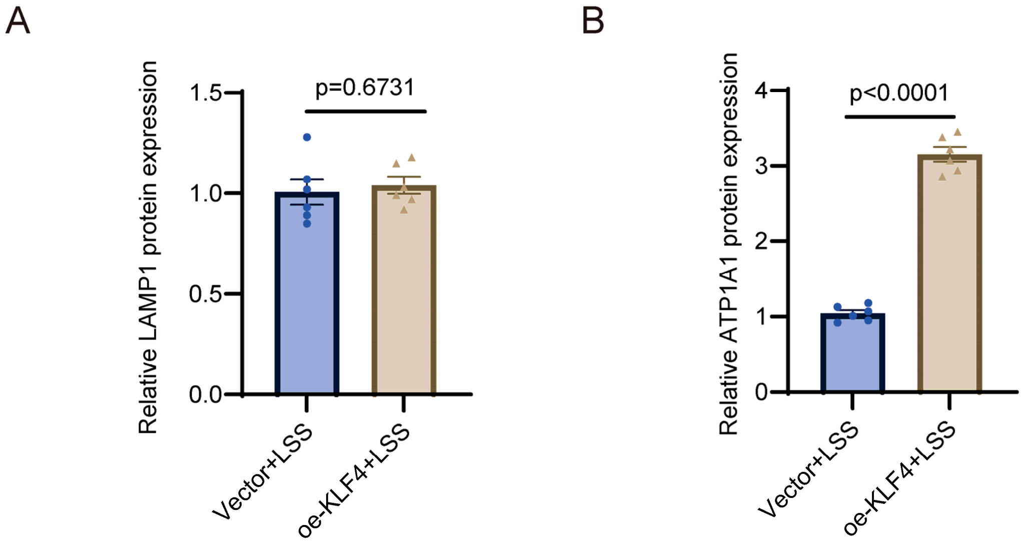

Figure S4 Quantification of lysosomal markers in HUVECs under LSS.

A, Densitometric quantification of LAMP1 expression in Vector+LSS and oe-KLF4+LSS HUVECs. B, Densitometric quantification of ATP1A1 expression in Vector+LSS and oe-KLF4+LSS HUVECs. HUVEC, human umbilical vein endothelial cell; LSS, low shear stress; oe-KLF4, adenovirus-mediated KLF4 overexpression; ATP1A1, Na^+/K^+-ATPase α 1 subunit; LAMP1, lysosomal-associated membrane protein 1; SEM, standard error of the mean. Statistical analyses were performed using unpaired two-tailed Student's t test, n=6 independent experiments.

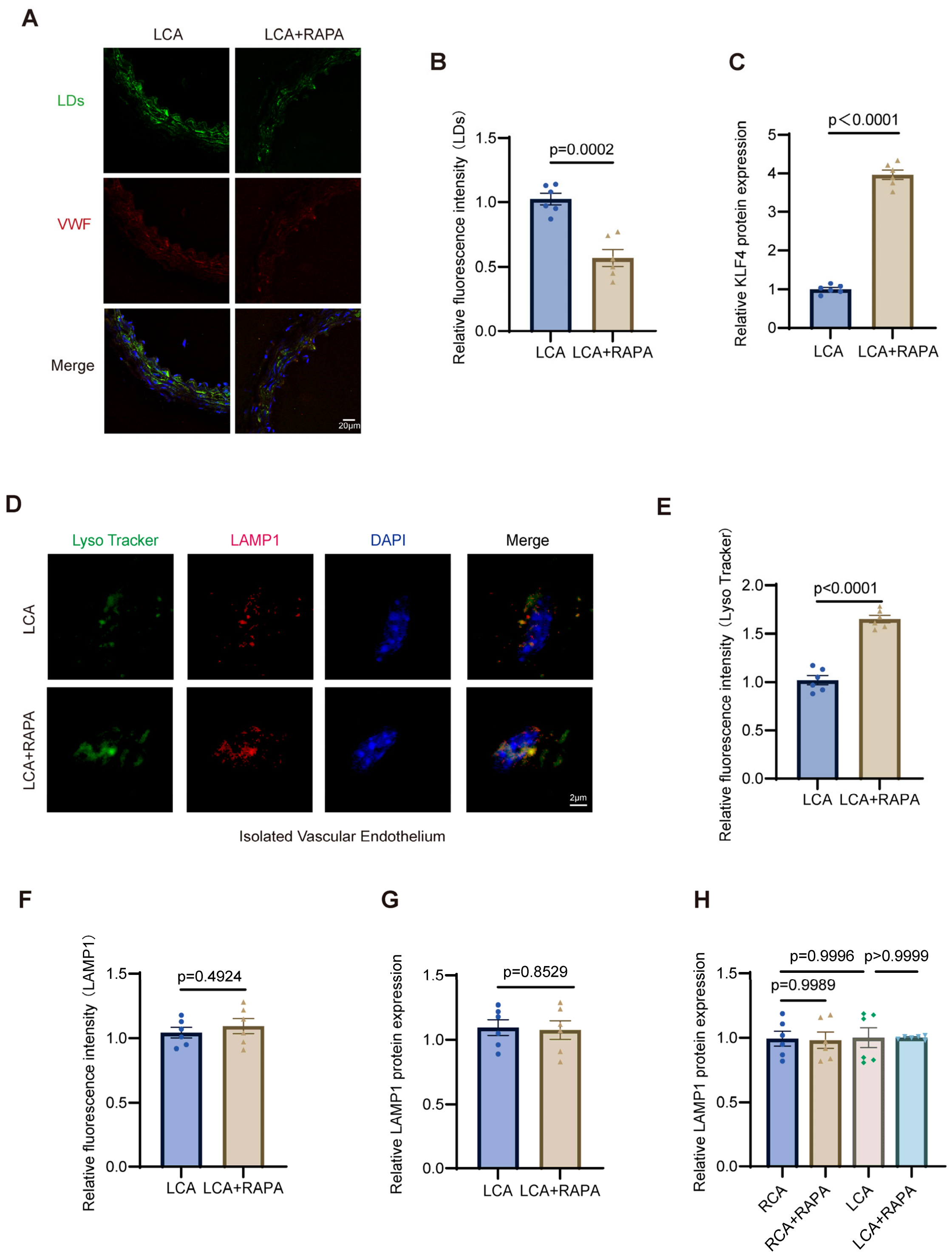

Figure S5 Rapamycin effects on endothelial lysosomal function and KLF4 expression in LCA.

A, Representative immunofluorescence images of frozen carotid artery sections from LCA and LCA+RAPA groups, showing VWF (red), LDs (green), and nuclei (DAPI, blue). B, Quantification of LD fluorescence intensity in LCA and LCA+RAPA frozen

sections. C, Densitometric quantification of KLF4 protein expression in LCA and LCA+RAPA groups (corresponding to Figure 5B). D, Enface immunofluorescence images of LCA and LCA+RAPA groups, showing LysoTracker (green), LAMP1 (red), and nuclei (DAPI, blue). E, Quantification of LysoTracker fluorescence intensity in LCA and LCA+RAPA groups. F, Quantification of LAMP1 fluorescence intensity in LCA and LCA+RAPA groups. G, Densitometric quantification of LAMP1 expression in LCA and LCA+RAPA groups (corresponding to Figure 5H). H, Densitometric quantification of LAMP1, LC3B-II/I ratio, and P62 in RCA, RCA+RAPA, LCA, and LCA+RAPA groups (corresponding to Figure 5J). LCA, left carotid artery; RCA, right carotid artery; HUVEC, human umbilical vein endothelial cell; LSS, low shear stress; PSS, physiological shear stress; LAMP1, lysosomal-associated membrane protein 1; LysoTracker, a lysosomal marker; SEM, standard error of the mean. All data are presented as mean ± SEM. Statistical analyses were performed using unpaired two-tailed Student's t test or two-way ANOVA followed by Tukey's multiple comparison test. n = 6 for animal experiments.

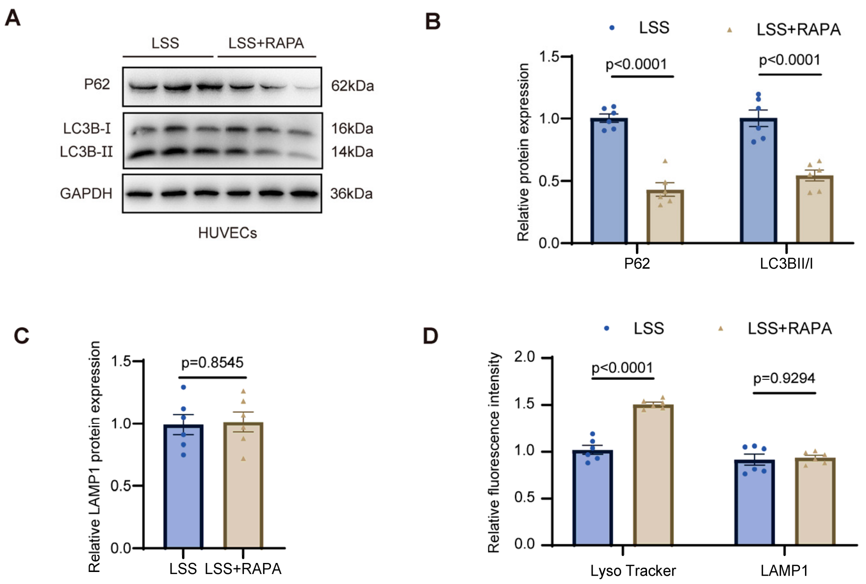

Figure S6 Restoration of lysosomal acidification–dependent lipophagy by rapamycin under LSS

A, Representative Western blot images of P62 and LC3B protein expression in LSS and LSS+RAPA groups. B, Densitometric quantification of P62 protein levels and LC3B-II/I ratio in LSS and LSS+RAPA groups. C, Densitometric quantification of LAMP1 protein expression corresponding to Figure 6I. D, Quantification of LAMP1 and LysoTracker fluorescence intensity in LSS and LSS+RAPA groups. LSS, low shear stress; RAPA, rapamycin; LC3B, microtubule-associated protein 1 light chain 3B; LAMP1, lysosomal-associated membrane protein 1; SEM, standard error of the mean. Quantitative data are presented in the form of mean ± SEM. Statistical analyses were performed using unpaired two-tailed Student's t test or two-way ANOVA

followed by Tukey's multiple comparison test. n = 6 independent experiments.

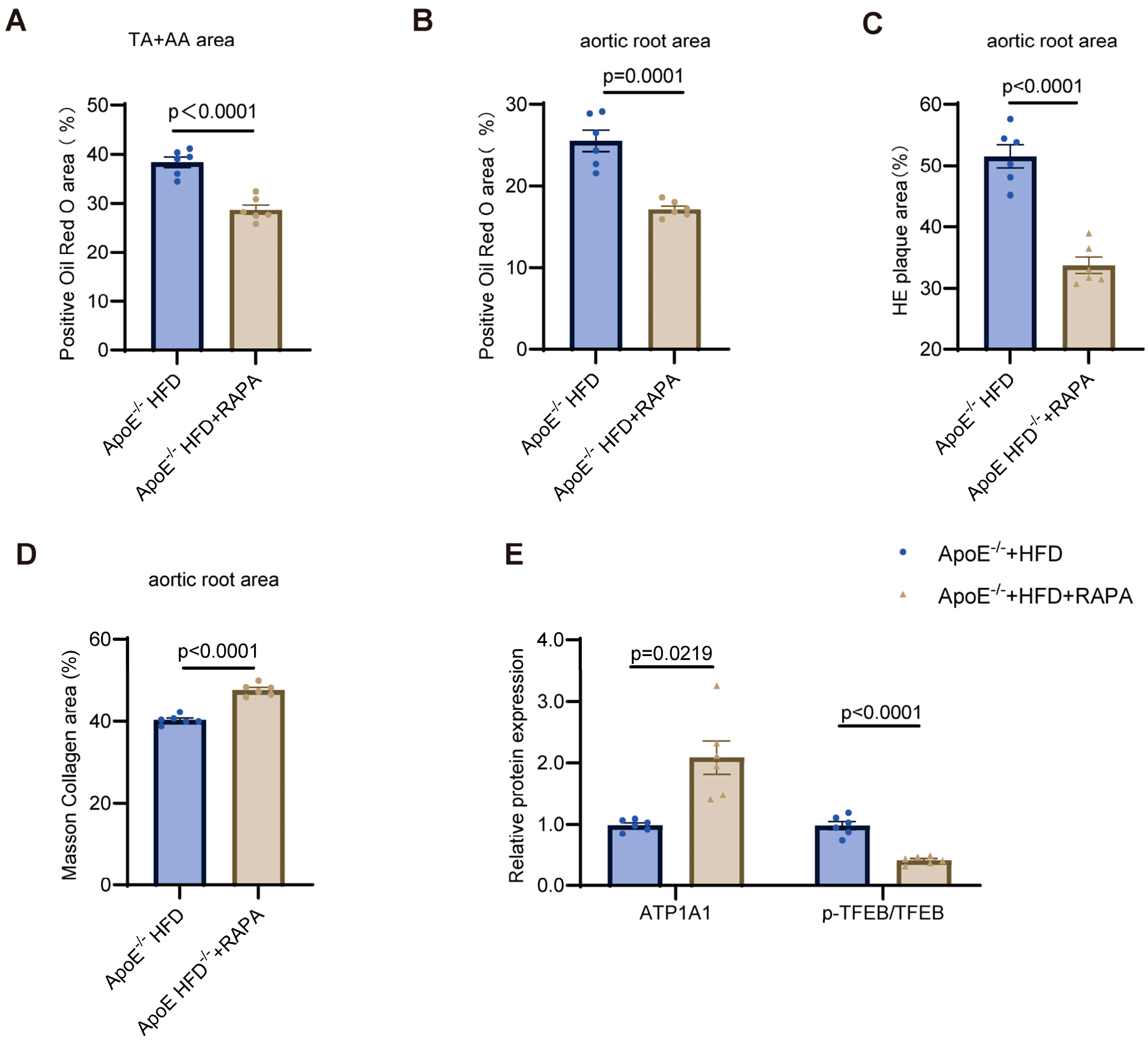

Figure S7 Quantification of rapamycin effects on aortic plaque and KLF4/TFEB axis.

A, Quantification of TA+AA positive area from Oil Red O staining (corresponding to Figure 7B-C). B, Quantification of aortic root positive area from Oil Red O staining. C, Quantification of HE positive area from HE staining. D, Quantification of Masson positive area from Massonstaining. D, Densitometric quantification of ATP1A1, p-TFEB/TFEB ratio, and total TFEB protein expression in AA regions (corresponding to Figure 7G).HFD, high-fat diet; RAPA, rapamycin; AA, aortic arch; TA, thoracic aorta; DAPI, 4′,6-diamidino-2-phenylindole; SEM, standard error of the mean. All data are presented as mean ± SEM. Statistical analyses were performed using unpaired two-tailed Student's t test or two-way ANOVA followed by Tukey's multiple comparison test. n = 6 for animal experiments.

Supplementary Table S1. Primers used in real-time RT-PCR analysis.

Gene	FW	REV
Human GAPDH	GTCAAGGCTGAGAACGGGAA	AAATGAGCCCCAGCCTTCTC
Human ATGL	AGGCTGGTGCCAAGTTCATT	AGACATTGGCCTGGATGAGC
Human CPT1	TTCAGTTCACGGTCACTCCG	TGACCACGTTCTTCGTCTGG
Human CPT2	CCGTCCACTTTGAGCACTCT	GCCATGGTACTTGGAGCACT
Human CD36	GGCTGTGACCGGAACTGTG	AGGTCTCCAACTGGCATTAGAA
Human KLF4	TCCAATTCGCTGACCCATCC	AAGAAGGTGGGGTGAGCATC
Mus musculus *KLF4*<br>G	AGGAACTCTCTCACATGAAGC	GGTCGTTGAACTCCTCGGTC
Mus musculus GAPDH	GGTCCCAGCTTAGGTTCATCA	CCTTTTGGCTCCACCCTTCA
Mus musculus ATGL	GCAATCTCTACCGCCTCTCG	TGGGTTGGTTCAGTAGGCCA
Mus musculus CPT1<br>G	ATGGAGAGCTTGCTACACGCA	CAGGGGTGACTGTGAACTGG
Mus musculus CPT2	AGCAGTGCTCTAAAGGCTGG	AAAGATGGAAGCTGGGCGAA
Mus musculus CD36	ATGGGCTGTGATCGGAACTG	TTTGCCACGTCATCTGGGTTT
