# Supplementary material for: Low Shear Stress Promotes Atherosclerosis by Mediating Pathological Accumulation of Endothelial Lipid Droplets via the KLF4/TFEB/ATP1A1 Axis
Source: J Cardiovasc Dev Dis. 2026 May 15;13(5):213. doi: 10.3390/jcdd13050213 (PMC13207403; doi:10.3390/jcdd13050213)
Supplement: Supplementary file 1 [file jcdd-13-00213-s001.zip › Supplementary File S3_ARRIVE Essential 10.pdf]

ARRIVE Essential 10

1. Study design

Explanation: This study employed multiple parallel experimental designs to investigate the role of low shear stress and the KLF4/TFEB/ATP1A1 axis in endothelial lipid metabolism and atherosclerosis.

Study 1: Diet-induced atherosclerosis model in ApoE$^{-/-}$ mice

To investigate the effects of low shear stress on regional lipid deposition in a classic mouse model of atherosclerosis, ApoE$^{-/-}$ male mice were randomly assigned to two dietary groups[49]:

ApoE$^{-/-}$ Chow group: standard chow diet (15 kcal% fat; Research Diets, D11112201)

ApoE$^{-/-}$ HFD group: high-fat diet (40 kcal% fat + 1.25% cholesterol; Research Diets, D12108C)

Mice were fed for 12 weeks to induce atherosclerosis.

For regional hemodynamic comparison, two anatomical segments were analyzed from each animal:

Aortic arch (AA): representative low shear stress (LSS) region

Thoracic aorta (TA): representative physiological shear stress (PSS) region

The TA served as an internal hemodynamic control.

Study 2: Partial carotid ligation model in C57BL/6 mice

To establish a disturbed-flow–induced LSS model independent of hyperlipidemia, male C57BL/6 mice underwent left carotid artery (LCA) partial ligation surgery[17].

Low shear stress was induced in vivo using a partial ligation model of the left common carotid artery (LCA). Three of its four branches—the external carotid artery (ECA), internal carotid artery (ICA), and occipital artery (OA)—were ligated, leaving the superior thyroid artery (STA) patent. The right common carotid artery (RCA) served as an internal control.

Internal control: right carotid artery (RCA,PSS region)

Experimental vessel: LCA (LSS region)

Study 3: LSS model with pharmacological intervention (C57BL/6 background)

C57BL/6 mice underwent partial ligation of the left carotid artery (LCA) as described above, followed by intraperitoneal injections of either RAPA or an equivalent volume of vehicle control[37].

Control: LCA +PBS

Experimental vessel: LCA + Rapamycin (RAPA) treatment

At study endpoint, the ligated LCA segments were harvested for analysis.

Study 4: Atherosclerosis model with pharmacological intervention (ApoE$^{-/-}$ background)

To evaluate the therapeutic potential of RAPA in the context of diet-induced atherosclerosis, ApoE$^{-/-}$ mice were first maintained on a high-fat diet (HFD; 40 kcal% fat and 1.25% cholesterol; Research Diets, D12108C). Subsequently, mice were administered RAPA (5 mg/kg/day) via intraperitoneal injection for a duration of 4 weeks, while the corresponding control group received an equal volume of vehicle[23].

Control: ApoE$^{-/-}$ HFD

Experimental group: ApoE$^{-/-}$ HFD+RAPA

were harvested separately for regional analysis: Aortic arch (AA; LSS region) Thoracic aorta (TA;

PSS region)

2. Sample size

The sample size (n= 6 per group) was determined based on a power analysis (α= 0.05, Power = 0.80) and is consistent with the AHA scientific statement on animal atherosclerosis studies, which recommends this range as sufficient to detect statistically significant differences in lesion area within the $ApoE^{-/-}$ mouse model while adhering to the ethical principle of reducing animal use [**Error! Reference source not found.**]. For the construction of the LSS model, C57BL/6 mice underwent partial ligation of the left carotid artery (LCA). Based on a power analysis (α= 0.05, Power = 0.80) and our prior experience with this surgical model, an initial sample size of n=8 was assigned to each group to account for potential procedure-related mortality, thereby ensuring a minimum of n=6 surviving animals per group for robust statistical analysis of regional lipid deposition and vascular remodeling.

3. Inclusion and exclusion criteria

a. Inclusion and exclusion criteria were established a priori. Animals were included in the final analysis if they successfully completed the designated treatment period and the surgical site showed no signs of infection.

b. While no animals in the $ApoE^{-/-}$ groups died, several mice in the LCA partial ligation groups were excluded from the study due to anesthesia complications, surgical trauma, or unexpected post-operative mortality[50]. Specifically, individuals that did not survive the initial 24-hour post-operative period were removed from the dataset to ensure the integrity of the LSS model[**Error! Reference source not found.**].

c. After these exclusions, an exact value of n=6 per group was maintained for all subsequent histological and biochemical analyses.

4. Randomisation

Eight-week-old male C57BL/6J and $ApoE^{-/-}$ mice (weighing 22–25 g) were obtained from the Experimental Animal Center of Nanjing Medical University. To minimize selection and environmental bias, a rigorous randomization protocol was implemented for all $ApoE^{-/-}$ and C57BL/6J mice. Using a computer-generated random number table (Excel 2019, Microsoft), each mouse was assigned a unique identification number and subsequently allocated to the Control, Model, or Treatment (Vehicle or RAPA) groups[51]. To further mitigate potential environmental influences, cage allocation was also randomized across the animal facility. This systematic randomization procedure ensured that all experimental units were distributed across treatment arms without investigator-induced bias.

5. Blinding

To ensure objectivity throughout the study, blinding was rigorously maintained at all experimental stages. Although the primary investigators were not blinded during group allocation and experimental procedures to ensure accurate administration of RAPA or high-fat diet, outcome assessments were conducted in a blinded manner. Specifically, all subsequent analyses， including histological plaque quantification, immunofluorescence analysis, Western blot densitometry, and collagen content evaluation， were carried out by investigators who were unaware of the treatment

assignments. To further minimize observer bias during data analysis, all samples and images were labeled with coded identifiers. The master key linking these codes to the experimental groups was concealed from the investigators performing the analyses and was only revealed after the statistical analyses were fully completed.

6. Outcome measures

Primary outcomes.

The primary outcome measure was atherosclerotic plaque burden in regions exposed to low shear stress. Plaque area was quantified by Oil Red O staining using both enface LDs analysis of the entire aorta and cross-sectional analysis where applicable. Regional plaque burden was compared between the AA and the TA region in $ApoE^{-/-}$ mice, as well as between the ligated LCA and the contralateral RCA in the partial ligation model. Quantification was performed using standardized image analysis software and expressed as percentage of lesion area relative to total vessel area. In addition, lysosomal acidification and endothelial lipid droplet accumulation were assessed as key functional readouts of endothelial lipid metabolic homeostasis. Lysosomal function was evaluated using pH-sensitive fluorescent probe Lyso-racker, and lipid droplets were quantified by BODIPY dye. The primary outcome (plaque area in low shear stress regions) was used to guide biological effect estimation and sample size determination.

Secondary outcomes.

Secondary outcome measures were designed to elucidate the molecular mechanisms underlying shear stress–dependent lipid metabolic regulation. Expression levels of KLF4, TFEB (including nuclear localization), and ATP1A1 were assessed by Western blotting, immunofluorescence, and/or quantitative PCR. Autophagic activity was evaluated by measuring LC3-II/I ratio and p62 protein levels to determine autophagic flux and lipophagy efficiency. Plaque composition was further characterized by collagen deposition using Masson's trichrome staining to assess indices of plaque stability. All outcome measures were predefined prior to study initiation, and no modifications to primary or secondary endpoints were made during the course of the study.

7. Statistical methods

This study used GraphPad Prism 8.0 software (GraphPad Software, San Diego, USA) for statistical analysis of data. First, data normality was tested. Quantitative data with a normal distribution were expressed as mean ± standard error of the mean (SEM). For data without a normal distribution, results were expressed as median (interquartile range) and were log-transformed before further analysis. Data normality was assessed using the Shapiro-Wilk test, and homogeneity of variance was evaluated with the Brown-Forsythe test. If data were not normally distributed, the Kruskal-Wallis non-parametric test was employed. A one-sample t-test was used to determine whether the mean of a sample was significantly different from a known population mean (such as 0), and an unpaired two-tailed Student's t test was used for comparisons between two independent groups. For multiple-group comparisons, one-way analysis of variance (ANOVA) followed by Bonferroni's post-hoc test was applied for single-factor designs, whereas two-way ANOVA followed by Tukey's multiple comparison test was used for two-factor designs. All tests were two-tailed, and a $P < 0.05$ was considered statistically significant.

8. Experimental animals

Male Apolipoprotein E–deficient ($ApoE^{-/-}$) mice on a C57BL/6J (8 weeks old) background and wild-type C57BL/6J mice (8 weeks old) were obtained from the Animal Laboratory of Nanjing Medical University. All mice were housed under specific pathogen–free (SPF) conditions and acclimatized for at least one week prior to experimental procedures. $ApoE^{-/-}$ mice were homozygous for the targeted deletion and were confirmed by the supplier. At the start of the study, mice weighed approximately 22–25 g and exhibited no signs of illness. No animals had undergone prior experimental manipulation before enrollment in this study.

9. Experimental procedures

Animals were housed in a temperature-controlled room (22 ± 2°C) with a 12-hour light/dark cycle and had free access to food and water. All procedures were performed in accordance with institutional guidelines.

Study 1: Diet-induced atherosclerosis model

To establish diet-induced atherosclerosis, male $ApoE^{-/-}$ mice (8 weeks old) were randomly assigned to receive either a standard chow diet (15 kcal% fat; Research Diets, D11112201) or a high-fat diet (HFD; 40 kcal% fat supplemented with 1.25% cholesterol; Research Diets, D12108C) for 12 weeks. The 12-week feeding period was selected based on established models demonstrating robust plaque development in $ApoE^{-/-}$ mice under these conditions[52]. At the end of the feeding period, mice were euthanized and the entire aorta was carefully dissected under a stereomicroscope. The AA and TA were separated for regional analysis. This regional comparison was performed to investigate hemodynamic-dependent differences in plaque burden and endothelial lipid metabolism.

Study 2: Partial carotid ligation model of low shear stress

To generate disturbed flow in vivo, partial ligation of the left common carotid artery (LCA) was performed in 8-week-old male C57BL/6J mice under inhalational anesthesia. Through a midline neck incision, three of the four distal branches of the LCA—the external carotid artery, internal carotid artery, and occipital artery—were ligated using 6-0 silk sutures, while the superior thyroid artery was left patent to maintain residual flow. The right common carotid artery (RCA) remained untouched and served as an internal physiological control. This procedure induces sustained low and oscillatory shear stress in the LCA without systemic lipid alteration. Mice were monitored during the postoperative recovery from anesthesia. The success of the procedure was determined by the visual confirmation of blood flow interruption under a stereomicroscope and the successful recovery of the mice from anesthesia. Following the procedure, mice were maintained for the designated experimental duration prior to tissue collection[13].

Study 3: Rapamycin intervention

To evaluate the therapeutic potential of rapamycin (RAPA), $ApoE^{-/-}$ mice fed HFD or C57BL/6J mice subjected to partial carotid ligation were administered rapamycin (5 mg/kg/day) via intraperitoneal injection for 4 consecutive weeks[22]. Rapamycin was dissolved in vehicle solution, and control animals received an equal volume of vehicle following the same schedule. Injections were performed once daily at consistent times to minimize circadian variability. The selected dose and treatment duration were based on prior studies demonstrating effective mTOR

inhibition and modulation of atherosclerotic progression in mice[53]. At the completion of treatment, animals were euthanized and vascular tissues were harvested for histological, biochemical, and molecular analyses.

Tissue collection and processing

At the designated endpoints, mice were euthanized under deep anesthesia. To ensure a clean field for analysis, we transcardially perfused the vascular system with ice-cold PBS to clear any residual blood. The aorta and carotid arteries were then meticulously dissected; depending on the planned downstream assays, these tissues were either fixed, immediately processed for live-cell staining, or snap-frozen in liquid nitrogen. We quantified the atherosclerotic burden using Oil Red O staining. For molecular insights, tissues were homogenized for protein or RNA extraction, while for immunofluorescence, vessels were embedded in OCT and sectioned following standard histological protocols.

10. Result

Quantitative data with a normal distribution were expressed as mean ± standard error of the mean (SEM). For data without a normal distribution, results were expressed as median (interquartile range) and were log-transformed before further analysis. Exact p-values are indicated in the figures and were calculated using the appropriate statistical tests.

Ethical statement

实验动物福利伦理审查同意书

Affidavit of Approval of IACUC

申请编号	13197	批准编号 Approval No.	IACUC-2007012

本《动物实验方案》经过实验动物伦理委员会审核，符合动物保护、动物福利和伦理原则，符合国家实验动物福利伦理的相关规定。方案的相关信息如下：

The animal use protocol listed below has been reviewed and approved by Institutional Animal Care and Use Committee（IACUC）

实验名称 Protocol Title	流体剪切力调控自噬体和 Weibel Palade小体互作的研究<br>Study on the interaction between autophagosomes and Weibel Palade bodies regulated by fluid shear stress				
申请人姓名 Applicant	李慧<br>Li Hui	职称/学位 Title/Degree	硕士研究生<br>master degree candidate	邮箱 Email	18862188294@163.com
实验负责人 Principle Investigator	张俊霞<br>Zhang Junxia	职称/学位 Title/Degree	博士<br>Doctor	邮箱 Email	15850770739@126.com
院系（部门） Department	南京医科大学附属南京医院<br>Nanjing First Hospital, Nanjing Medical University			申请日期 Application Date	2020-06-29
拟实验时间 Period of Protocol	2020-08-30 - 2022-06-30	实验动物使用许可证 Number of Animal Use Permit		SYXK(苏)2016-0006	
审核意见 Results of Inspection	符合动物福利伦理要求，可以进行实验。 Agree				
兽医师 Chief Veterinary Officer	张爱华 Zhangaihua	张爱华 Zhang Aihua		日期 Date	2020.7.13

南京医科大学实验动物福利伦理委员会

Institutional Animal Care and Use Committee of NMU

主席(chairman)：施爱民 shiaimin 签名(signature)：施爱民 Shi [illegible]

日期(Date)：2020-07-13 2020.7.13
